# Bridging across patient subgroups in phase I oncology trials that incorporate animal data

**DOI:** 10.1177/0962280220986580

**Published:** 2021-01-27

**Authors:** Haiyan Zheng, Lisa V Hampson, Thomas Jaki

**Affiliations:** 1 Population Health Sciences Institute, Newcastle University, Newcastle upon Tyne, UK; 2 Department of Mathematics and Statistics, Lancaster University, Lancashire, UK; 3 Advanced Methodology and Data Science, Novartis Pharma AG, Basel, Switzerland; 4 MRC Biostatistics Unit, University of Cambridge, Cambridge, UK

**Keywords:** Bayesian hierarchical models, bridging, historical data, phase I clinical trials, robustness

## Abstract

In this paper, we develop a general Bayesian hierarchical model for bridging across patient subgroups in phase I oncology trials, for which preliminary information about the dose–toxicity relationship can be drawn from animal studies. Parameters that re-scale the doses to adjust for intrinsic differences in toxicity, either between animals and humans or between human subgroups, are introduced to each dose–toxicity model. Appropriate priors are specified for these scaling parameters, which capture the magnitude of uncertainty surrounding the animal-to-human translation and bridging assumption. After mapping data onto a common, ‘average’ human dosing scale, human dose–toxicity parameters are assumed to be exchangeable either with the standardised, animal study-specific parameters, or between themselves across human subgroups. Random-effects distributions are distinguished by different covariance matrices that reflect the between-study heterogeneity in animals and humans. Possibility of non-exchangeability is allowed to avoid inferences for extreme subgroups being overly influenced by their complementary data. We illustrate the proposed approach with hypothetical examples, and use simulation to compare the operating characteristics of trials analysed using our Bayesian model with several alternatives. Numerical results show that the proposed approach yields robust inferences, even when data from multiple sources are inconsistent and/or the bridging assumptions are incorrect.

## 1 Introduction

Bridging strategies are increasingly being used in the paradigm of global drug development^1–4^ to minimise duplication of clinical research without disregarding heterogeneity between patient groups. Bridging studies may be conducted in a new geographic region to evaluate whether a medicine’s performance (typically efficacy) is consistent with its performance in other parts of the world where it has been approved based on a complete development program. The International Conference on Harmonisation (ICH) E5 Guideline^5,6^ discusses whether and when trial data generated in an ‘original’ region can be leveraged to support the evaluation of drug activities in a new region where a sponsor is seeking registration. The degree of borrowing, ranging from none to full, is a matter of negotiation between the sponsor and the local health authority. By avoiding the unnecessary replication of evidence, bridging strategies can mitigate the drug lag problem^7–9^ and expedite patient access to new medicines.

Over the past few decades, the Pharmaceuticals and Medical Devices Agency (PMDA) in Japan has promoted synchronisation of clinical drug development in Japan and other countries.^10^ The agency encourages domestic sponsors to participate in global phase I dose-finding studies in oncology, which has led to a number of early phase bridging studies.^[Bibr bibr11-0962280220986580]^ It was further contended that phase I trials in Japan could be carried out in similar times as those in the west, based on the finding of small between-region heterogeneity in the toxicity profile of single agents, as 54 phase I oncology trials conducted at the National Cancer Center Hospital in Japan between 1995 and 2012 had been reviewed.^11^ In this paper, we will focus on the design and analysis of phase I bridging studies, which aim to support estimation of the maximum tolerated dose (MTD) in a new geographic region or patient subgroup of a previously studied disease indication. We want to leverage the dose–toxicity data available in relevant, already studied populations, without neglecting possible heterogeneity stemming from *intrinsic* factors such as a patient’s genetic make-up, and/or *extrinsic* factors such as diagnostic criteria and environmental exposures. Wider application of the proposed Bayesian model can also include, for example, phase I trials evaluating the toxicity profile of a given treatment for multiple disease subtypes.

Several model-based designs have been proposed for phase I clinical trials to account for potentially different safety profiles of a new medicine in various patient subgroups. Liu et al.^12^ develop a bridging continual reassessment method (CRM) procedure that uses the dose–toxicity data from a completed historical trial to generate multiple sets of ‘skeleton’ probabilities for a new trial in a different geographic region, with the most plausible set of skeleton probabilities weighted favourably through the Bayesian model averaging.^13^ Takeda and Morita^14^ present a Bayesian dose-escalation procedure which dynamically leverages information from a historical study. Specifically, before the new trial begins, historical trial data are used to formulate a weakly informative prior for the parameter of a dose–toxicity model employed by the CRM for dose recommendations; so-called weakly informative because the prior effective sample size^15^ is considerably smaller than the anticipated sample size of the new trial. Historical and new trial data are then linked through a ‘historical-to-current’ parameter, which reflects the degree of agreement between the studies.

Alternatively, relevant ‘complementary-data’ (or co-data for short)^16^ can be leveraged from phase I clinical trials run concurrently with the trial of interest, or from commensurate patient subgroups enrolled in the same trial. O’Quigley et al.^17^ propose a two-sample CRM to draw inferences about the MTD appropriate for each of the two non-overlapping subgroups of patients. The dose–toxicity curves are modelled through a pair of parameters, one of which represents information common to both subgroups and the other, as a ‘shift parameter’, for heterogeneity of the second subgroup in relation to the first. O’Quigley and Iasonos^18^ discuss theoretical properties of this bridging model when the shift parameter is discrete, allowing for the recommended dose in the second subgroup to be one or two dose levels away from the estimate in the first. Wages et al.^19^ extend this CRM-type shift model to account for uncertainty about the true shifts and design a phase I/II trial of stereotactic body radiation therapy, where the dose–response relationship may present as non-monotonic.

To date, designs for phase I bridging studies have focused on co-data from trials conducted under similar circumstances, for example, studies evaluating a different yet relevant patient subgroup. However, preliminary data from animal toxicology studies will also be available, as is required by regulatory authorities.^20^ It is appealing to use the animal and external human trial data, in addition to any human trial data from relevant patient subgroups, so that dose recommendations at early stages of the phase I trial can be informed by all relevant evidence.^21,22^ The challenge is to properly link the dose–toxicity models for different animal species and human subgroups. In situations of strong differences between toxicity profiles, the co-data should be quickly discounted from the analysis of the new phase I clinical trial.

Zheng et al.^21^ propose a robust Bayesian hierarchical model to leverage data from multiple animal species in a phase I oncology trial which will be performed in a homogeneous patient group, to support the interim and final dosing recommendations. In this paper, we extend their approach to accommodate the case that the study population is made of heterogeneous patient subgroups. The robust extention proposed in this paper can therefore augment a phase I bridging trial with co-data, which may comprise (i) data from completed preclinical animal studies and/or (ii) concurrent external data from either completed or ongoing trials conducted in related patient subgroups (e.g. patients from other geographic regions). When the intrinsic and extrinsic factors arising from ethnicity would result in heterogeneous dose–toxicity relationships, our model will estimate the subgroup-specific MTDs.

The remainder of this paper is structured as follows. In Section 2, we develop a robust Bayesian hierarchical random-effects model leveraging data from both animal studies and related human subgroups, to support the analysis of a new phase I oncology trial. In Section 3, we illustrate the use of the proposed methodology for improved decision making with various hypothetical data examples. In Section 4, we perform a simulation study to compare the operating characteristics of dose-escalation trials driven by the proposed model with several alternatives. Finally, we draw conclusions and look towards future research in Section 5.

## 2 Bayesian hierarchical model for animal data and heterogeneous human data

In this section, we generalise the Bayesian model of Zheng et al.^21^ to leverage available animal data and dose–toxicity data from different human subgroups into new phase I clinical studies.

Suppose that at the time of planning a phase I clinical trial, *M* preclinical studies have been performed in *K* animal species, labelled $S_{1},\ldots ,S_{K}$ . For i=1,…,M , animal study *i* tested a total of *J_i_* doses contained in set $D_{i}=\{d_{i1},\ldots ,d_{iJ_{i}};d_{it_{1}}\leq d_{it_{2}}\text{ for }1\leq t_{1}\leq t_{2}\leq J_{i}\}$ . On receiving dose $d_{ij}\in D_{i}$ , an animal experiences a dose-limiting toxicity (DLT) with probability *p_ij_* and no DLT with probability $1-p_{ij}$ . Let *n_ij_* and *r_ij_* be the number of animals that received dose *d_ij_* and the number that experienced a DLT, respectively. We assume a monotonic increasing relationship between *p_ij_* and *d_ij_*, which can be adequately described by a two-parameter logistic regression model^23,24^

(1) $$\begin{array}{l} r_{ij}|p_{ij},n_{ij}∼\text{Binomial}(p_{ij},n_{ij}),\text{ for }j=1,\ldots ,J_{i},\\ \text{logit}(p_{ij})=\theta_{1i}+\exp(\theta_{2i})\log(\delta_{A_{i}}d_{ij}/d_{\text{Ref}}) \end{array}$$

where $\delta_{A_{i}}$ is a translation parameter mapping animal doses onto an equivalent human dosing scale. Zheng et al.^21^ propose placing a tailored log-normal prior on $\delta_{A_{i}}$ to account for the intrinsic differences between the toxicity of the drug in animal species $A_{i}\in \{S_{1},\ldots ,S_{K}\}$ and humans. Thus, model parameters $\theta_{i}=(\theta_{1i},\theta_{2i})$ describe the dose–toxicity relationship on an equivalent human dosing scale. In Model (1), $d_{\text{Ref}}$ is a reference dose invariant across all dose–toxicity studies, which is often chosen to be the most probable level of the human MTD.

Random-effects distributions are stipulated on the second level of the hierarchical model to enable information sharing between animal studies of the same species

(2) $$\theta_{i}|\mu_{A_{i}},\Psi ∼\text{BVN}(\mu_{A_{i}},\Psi )$$

with

$$\begin{array}{l} \mu_{A_{i}}=\left(\begin{array}{l} \mu_{1A_{i}}\\ \mu_{2A_{i}} \end{array}\right)\;\text{ and }\;\Psi =\left(\begin{array}{ll} \tau_{1}^{2} & \rho \tau_{1}\tau_{2}\\ \rho \tau_{1}\tau_{2} & \tau_{2}^{2} \end{array}\right) \end{array}$$

for $A_{i}\in \{S_{1},\ldots ,S_{K}\}$ . Variances in Ψ reflect between-study heterogeneity within an animal species. A ‘supra-species’ random effects distribution is introduced to facilitate borrowing of information across different animal species. That is, for species $S_{k},\;k=1,\ldots ,K$

(3) $$\mu_{S_{k}}|m,\Sigma ∼\text{BVN}(m,\Sigma )$$

with

$$\begin{array}{l} m=\left(\begin{array}{l} m_{1}\\ m_{2} \end{array}\right)\;\text{ and }\;\Sigma =\left(\begin{array}{ll} \sigma_{1}^{2} & \kappa \sigma_{1}\sigma_{2}\\ \kappa \sigma_{1}\sigma_{2} & \sigma_{2}^{2} \end{array}\right) \end{array}$$

This ‘supra-species’ random-effects distribution accounts for the differences between toxicity parameters in different species which are not addressed by the translation parameters $\delta_{S_{1}},\ldots ,\delta_{S_{K}}$ .

We now focus on modelling the human toxicity data that will be collected from different human subgroups. Suppose there are a total of *L* predefined, non-overlapping human subgroups and one trial only is performed in each subgroup. To distinguish from the notation used for animal studies, we let ℓ=1,…,L index the new human trials wherein doses in $D_{\ell }=\{d_{\ell 1},\ldots ,d_{\ell J_{\ell }};d_{\ell t_{1}}\leq d_{\ell t_{2}}\text{ for }1\leq t_{1}\leq t_{2}\leq J_{\ell }\}$ are to be evaluated, and let $\gamma_{\ell }=(\gamma_{1\ell },\gamma_{2\ell })$ be the counterpart of $\theta_{i}$ . That is, $\gamma_{\ell }$ underpins the dose–toxicity relationship in human subgroup ℓ . Model (1) is also applicable to describe the human toxicity data, only that we will set the animal-to-human translation parameter $\delta_{A_{i}}=1$ and introduce a subgroup-specific parameter denoted by $\epsilon_{\ell }$ for subgroup ℓ . For a phase I clinical trial ℓ=1,…,L , the human toxicity data can be described by

(4) $$\begin{array}{l} r_{\ell j}|p_{\ell j},n_{\ell j}∼\text{Binomial}(p_{\ell j},n_{\ell j}),\text{ for }j=1,\ldots ,J_{\ell },\\ \text{logit}(p_{\ell j})=\gamma_{1\ell }+\exp(\gamma_{2\ell })\log(\epsilon_{\ell }d_{\ell j}/d_{\text{Ref}}) \end{array}$$

where $d_{\text{Ref}}$ is the same reference dose used in Model (1) and $\epsilon_{\ell }$ adjusts for the differences in toxicity arising from the intrinsic and/or extrinsic factors across human subgroups. In particular, this parameterisation maps the ‘average’ human dosing scale to the dose–toxicity profile of a particular subgroup. We regard each $\epsilon_{\ell }$ as a random variable, on which we place a truncated normal prior distribution centred at 1 with variance $\nu_{\ell }^{2}$ for positive real numbers only, due to the use of a logrithm transformation of the scaled doses.

Here, instead of the log-normal priors which we specify for the animal-to-human translation parameters, we consider priors with a mode of 1 for $\epsilon_{\ell }$ : it is reasonable to assume that dose–toxicity data collected from each human subgroup are on the ‘average’ human dosing scale already. The cases, $0<\epsilon_{\ell }<1$ and $\epsilon_{\ell }>1$ , correspond to scenarios that the drug (at the same dose) is less or more toxic in subgroup ℓ than on average in humans, respectively. To ensure symmetry about the mode of 1, these normal priors are truncated to fall within (0, 2); formally, these are $N(1,\nu_{\ell }^{2})I(0\leq \epsilon_{\ell }\leq 2)$ , with *I*(*a*) being the indicator function that *I*(*a*)* *=* *1 if *a* is true. For example, one could place a truncated normal prior $N(1,0.255^{2})I(0\leq \epsilon_{\ell }\leq 2)$ on each $\epsilon_{\ell }$ . Under this choice, 95% prior probability mass is concentrated on the interval [0.5, 1.5], meaning that the region-specific MTDs, if divergent, have less than 0.5-fold change between one another. The variance $\nu_{\ell }^{2}$ could be increased if even larger differences in toxicity across regions are considered plausible.

For simplicity, we assumed that one trial only had been undertaken per human subgroup. Following Neuenschwander et al.,^16^ our model accommodates two exchangeability scenarios, along with one non-exchangeability scenario, for $\gamma_{1},\ldots ,\gamma_{L}$ , respectively. The former precisely includes, (a) parameters of animal and human dose–toxicity relationships are exchangeable with each other; and (b) human dose–toxicity parameters are exchangeable only with those of other human subgroups. For human subgroup ℓ=1,…,L , we stipulate that

- For k=1,…,K , with prior probability $w_{\ell S_{k}}$
$$\gamma_{\ell }|\mu_{S_{k}},\Psi ∼\text{BVN}(\mu_{S_{k}},\Psi );$$

This represents exchangeability between $\gamma_{\ell }$ and the study-specific parameters relating to animal species *S_k_*.

- With prior probability $w_{\ell H}$
(5) $$\gamma_{\ell }|\mu_{H},\Phi ∼\text{BVN}(\mu_{H},\Phi )$$

where

$$\begin{array}{l} \mu_{H}=\left(\begin{array}{l} \mu_{1H}\\ \mu_{2H} \end{array}\right)\;\text{ and }\;\Phi =\left(\begin{array}{ll} \tau_{3}^{2} & \eta \tau_{3}\tau_{4}\\ \eta \tau_{3}\tau_{4} & \tau_{4}^{2} \end{array}\right); \end{array}$$

so that $\gamma_{\ell }$ is exchangeable only with the study-specific parameters for other human subgroups. Here, Φ captures a combination of between-study and between human subgroup heterogeneity.

- With prior probability $w_{\ell R}=1-\sum_{k}w_{\ell S_{k}}-w_{\ell H}$
$$\gamma_{\ell }∼\text{BVN}(m_{0\ell },R_{0\ell })$$

so that $\gamma_{\ell }$ is non-exchangeable with any other dose–toxicity parameters.

The prior probabilities $w_{\ell S_{1}},\ldots ,w_{\ell S_{K}},\;w_{\ell H}$ and $w_{\ell R}$ need to be pre-specified, before the conduct of a phase I trial in subgroup ℓ . Input from translational scientists or pharmacologists will be invaluable. In particular, stipulating a large $w_{\ell S_{k}}$ or $w_{\ell H}$ reflects a high level of prior confidence in the relevance of data from animal species *S_k_* or in the bridging assumption. Modelling exercise could also be used as additional evidence to inform expert opinion. For example, determination of such prior probabilities $w_{\ell^{⋆}S_{1}},\ldots ,w_{\ell^{⋆}S_{K}},\;w_{\ell^{⋆}H}$ and $w_{\ell^{⋆}R}$ for a new trial in setup, labelled by $\ell^{⋆}$ , could be suggested by the posterior probabilities of exchangeability and non-exchangeability pertaining to the subgroups $\ell \neq \ell^{⋆}$ involved in the co-data, as the latter helps our understanding about the overall commonality (or conversely, heterogeneity) between toxicities in animals and humans.

The second ‘human only’ exchangeability distribution in our model has its own covariance matrix Φ . This is because the degree of heterogeneity between study-specific dose–toxicity parameters in humans may be quite different to the level of heterogeneity between study-specific parameters in animals or the variations across species, captured by Ψ and Σ, respectively. When animal data have very limited predictability of the human toxicity yet the human toxicity data between themselves share considerable commonality, our robust hierarchical model will lead to large posterior probabilities being attributed to the (K+1) th ‘human only’ exchangeability distribution. For additional robustness, the model assigns positive prior probability $w_{\ell R}$ to the case that $\gamma_{\ell }$ is not exchangeable with any other parameter vectors. When the dose–toxicity relationship of a human subgroup appears to be an outlier, that is, dissimilar to that of any other human subgroup or animal species, the parameters can be estimated based on their own independent prior $\text{BVN}(m_{0\ell },R_{0\ell })$ .

**Figure 1.**
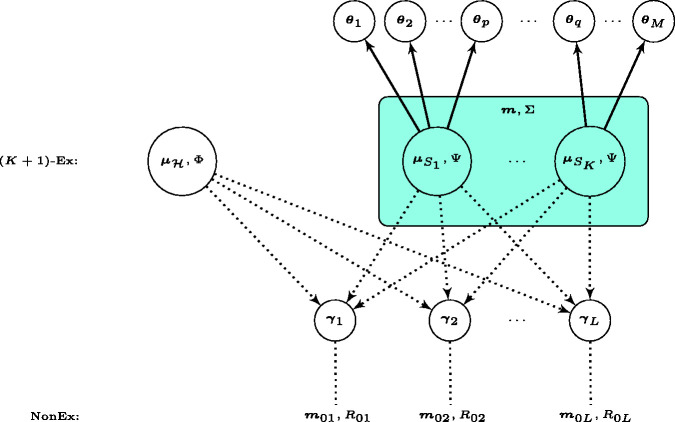
Diagram for the core of the proposed Bayesian hierarchical model. The solid (dotted) arrow suggests where a full (partial) exchangeability assumption holds. The possibility of non-exchangeability is enabled per human dose-toxicity parameter vector $\gamma_{\ell }$ , as suggested by the dotted verticle line.

We visualise the core of the proposed hierarchical model with a diagram in Figure 1, where (K+1) exchangeability distributions together with one non-exchangeability distribution are stipulated for each human dose–toxicity parameter vector $\gamma_{\ell },\;\ell =1,\ldots ,L$ . In this simplified setting, between-subgroup heterogeneity cannot be disentangled from between-trial heterogeneity. It is possible to expand our model to accommodate multiple trials per subgroup. Letting $i=1,\ldots ,M_{\ell }$ index a trial in subgroup ℓ=1,…,L , we may denote the trial-specific parameter vector by $\gamma_{\ell i}$ , and further assume that each set of $\gamma_{\ell 1},\ldots ,\gamma_{\ell n_{\ell }}$ are random samples drawn from their own ‘human only’ exchangeability distribution, say, BVN $\left(\mu_{H_{\ell }},\Phi \right)$ . The population means $\mu_{H_{1}},\ldots ,\mu_{H_{L}}$ could be assumed as exchangeable to enable sharing of information across the human subgroups. For robust inferences, the probability of exchangeability would be split to assume (a) $\gamma_{\ell i}|\mu_{S_{k}},\Psi ∼$ BVN( $\mu_{S_{k}},\Psi$ ) with probability $w_{\ell iS_{k}},\;k=1,\ldots ,K$ , and (b) $\gamma_{\ell i}|\mu_{H_{\ell }},\Phi ∼$ BVN( $\mu_{H_{\ell }},\Phi$ ) with probability $w_{\ell iH_{\ell }}$ . In the meanwhile, non-exchangeability distribution would remain specific to each parameter vector $\gamma_{\ell i}$ , that is, $\gamma_{\ell i}∼$ BVN( $m_{0\ell },R_{0\ell }$ ) with probability $w_{\ell iR}=1-\sum_{k}w_{\ell iS_{k}}-w_{\ell iH_{\ell }}$ for each individual trial *i* in subgroup ℓ .

To complete our Bayesian model, we now specify priors for other parameters. Weakly informative priors are placed on the hyperparameters of the random effects distributions in Models (3) and (5). The weakly informative priors used in subsequent sections are chosen so that each human toxicity risk $p_{\ell j}$ has a wide 95% prior credible interval.^25^ For the ‘supra-species’ population means $m=(m_{1},m_{2})$ , we set $m_{1}∼N(b_{1},s_{1}^{2})$ and $m_{2}∼N(b_{2},s_{2}^{2})$ . The same normal priors are used for $\mu_{1H}$ and $\mu_{2H}$ , respectively. Priors for the variance parameters should reflect opinion on the degree of between-source heterogeneity. Here, we propose setting

(6) $$\begin{array}{l} \tau_{1}∼HN(z_{1}),\;\tau_{2}∼HN(z_{2}),\;\tau_{3}∼HN(z_{3}),\;\tau_{4}∼HN(z_{4}),\\ \sigma_{1}∼HN(c_{1}),\;\sigma_{2}∼HN(c_{2}),\;\rho ∼U(-1,1),\;\kappa ∼U(-1,1),\;\eta ∼U(-1,1) \end{array}$$

where *HN*(*z*) denotes a half-normal distribution formed by truncating a normal distribution $N(0,z^{2})$ to fall within ( 0,∞ ). In Section 3 with hypothetical data scenarios, we will give an example of how to specify these hyper priors for robust inferences. The proposed robust Bayesian hierarchical model can be fitted using Markov chain Monte Carlo. The OpenBUGS^26^ code, together with R functions, for the implementation of our Bayesian analysis model is available through https://github.com/haiyanzheng/phaseI_bridging.

## 3 Illustrative example

In this section, we apply the robust Bayesian hierarchical model proposed in Section 2 to a hypothetical example informed by a real trial which aimed to characterise the toxicity profile of GSK3050002,^27^ an antibody for treating patients with psoriatic arthritis. The original trial enrolled a total of 49 human subjects exclusively in the United Kingdom. For illustration, we assume that two hypothetical phase I trials (labelled $T_{1}$ and $T_{2}$ ) are to be performed sequentially in two geographic regions, $R_{1}$ and $R_{2}$ , respectively, with trial $T_{1}$ performed first. The co-data for trial $T_{2}$ thus comprises data from trial $T_{1}$ and animal data, where available. The choice of animal species, animal doses and human doses for our numerical studies are informed by the real GSK phase I clinical trial. For present purposes, we assume the principal aim of these hypothetical trials is to estimate a region-specific MTD, defined as the dose associated with DLT risk of 25%.

### 3.1 Hypothetical preclinical data and predictive priors for human DLT risks

According to the protocol of GSK3050002,^28^ preclinical toxicity studies were performed in monkeys and rats. Moreover, monkeys were thought to be the most relevant animal species for predicting toxicity in humans. In the two real monkey studies, doses 1, 10, 30, 100 mg/kg were tested on 4–12 monkeys per dose group. From the trial protocol, it was not possible to identify what dose levels were used in rats, nor the exact number of rats treated, nor the number of toxicities observed. We therefore simulate plausible animal datasets based on the limited information available, and use these simulated data to obtain predictive priors for the human DLT risk at doses contained in the set $D_{\ell }=\{0.1,0.5,1,5,10,20\}$ mg/kg, which will be evaluated in trials $T_{1}$ and $T_{2}$ . The simulated animal data are represented in Figure S1 of the Web-based Supplementary Materials.

Throughout, we set $d_{\text{Ref}}=5$ mg/kg and use the priors as follows. Let $\mu_{1H},m_{1}∼N(-1.099,1.98^{2})$ and $\mu_{2H},m_{2}∼N(0,0.99^{2}),\;\sigma_{1}∼HN(1)$ and $\sigma_{2}∼HN(0.5)$ , and κ,η∼U(−1,1) . We assume moderate-to-substantial heterogeneity between studies in the same species, and therefore let $\tau_{1}∼HN(0.5),\tau_{2}∼HN(0.25)$ . Furthermore, we assume small-to-moderate heterogeneity between ethnic subgroups, captured by $\tau_{3}∼HN(0.25),\tau_{4}∼HN(0.125)$ . Here, we stipulate a half-normal prior *HN*(*z*) with smaller *z* for the slope than that for the intercept, because we think it is plausible that the slopes of dose–toxicity curves will be more similar than intercepts across studies, species and subgroups, respectively.^29^ Following Zheng et al.,^21^ we set $\delta_{\text{Rat}}∼LN(-1.820,0.323^{2})$ and $\delta_{\text{Monkey}}∼LN(-1.127,0.273^{2})$ to translate the animal data onto a common human scale.

**Figure 2.**
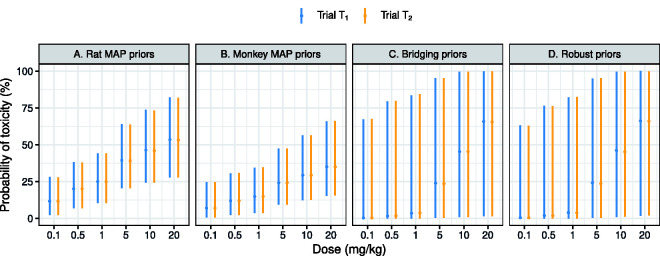
Summaries about the predictive priors for human toxicity, when using animal data from a single species (Panels A and B) or no animal data at all yet with a bridging assumption (Panel C) or without (Panel D). Medians together with 95% credible intervals of the marginal predictive priors are plotted.

For a robust inference under scenarios of data inconsistency, independent non-exchangeability distributions BVN $\left(m_{0\ell },R_{0\ell }\right)$ are specified for each $\gamma_{\ell }$ . Specifically, we set $m_{01\ell }∼N(-1.099,2^{2})$ and $m_{02\ell }∼N(0,1^{2})$ , with a zero correlation between $m_{01\ell }$ and $m_{02\ell }$ . By setting $w_{\ell S_{k}}=1$ and all other $w_{\ell S_{k\prime }}=0,\;k\prime \neq k$ , together with $w_{\ell H}$ and $w_{\ell R}$ as 0, the meta-analytic predictive (MAP) priors for $p_{\ell 1},\ldots ,p_{\ell J_{\ell }}$ are based on the animal data of a single species. For example, to see how human DLT risks may be predicted by the monkey data, we can fix $w_{\ell \text{Rat}}=0,\;w_{\ell \text{Monkey}}=1,\;w_{\ell H}=0,w_{\ell R}=0$ . When we set $w_{\ell R}=1$ and retain the rest as 0, no animal data will be used, nor are we making an assumption of bridging. Figure 2 summarises the MAP priors by source of information. Such summaries are useful to examine whether our Bayesian model can borrow (discount) information quickly from a particular species, given the data consistency (inconsistency).

As we can see, the rat and monkey data predict 1 mg/kg and 5 mg/kg as doses highly likely to result in a human DLT risk close to 25% in a human trial. After translation of the animal doses, rat data are mainly projected on the low doses of $D_{\ell }$ . Predictive priors obtained solely from rat data are thus more diffuse at high doses such as 10 mg/kg and 20 mg/kg, at which the monkey data in contrast have produced predictive priors for the DLT risks with narrower credible intervals. Patients recruited in regions $R_{1}$ and $R_{2}$ are predicted as having similar DLT risks based on the animal data. This is because for ℓ=1,2 , we specify the same prior probabilities $w_{\ell \text{Rat}},\;w_{\ell \text{Monkey}},\;w_{\ell H}$ and $w_{\ell R}$ , as well as the same truncated normal prior on $\epsilon_{\ell }$ , have been chosen for each human trial ℓ at the outset.

We obtain MAP priors for the DLT risk in humans, by allocating prior weights to different animal species on the basis of their *a priori* predictability of the human toxicity. For trial $T_{1}$ , we stipulate $w_{1\text{Rat}}=0.2,\;w_{1\text{Monkey}}=0.6,\;w_{1H}=0$ and $w_{1R}=0.2$ . No prior probability has been allocated to the exchangeability distribution for bridging across patient subgroups because trial $T_{2}$ has not yet started, meaning that the co-data for $T_{1}$ are exclusively from animal studies. We note this is the Bayesian model proposed by Zheng et al.,^21^ suitable for leveraging animal data to one homogeneous patient group. Figure S2 of the Supplementary Materials gives summaries of the MAP priors that robustly synthesise information across animal species to predict the human DLT risks. As soon as trial $T_{2}$ begins, the (K+1) th exchangeability component comes into play. For illustration, we set $w_{2\text{Rat}}=0.1,\;w_{2\text{Monkey}}=0.5,\;w_{2H}=0.2$ and $w_{2R}=0.2$ to leverage both animal data and the $T_{1}$ trial data, the human data from region $R_{1}$ , into trial $T_{2}$ . During the conduct of trial $T_{2}$ , the specification for prior probabilities $w_{1S_{k}}$ and $w_{1R}$ remain unchanged. In other words, no data from trial $T_{2}$ will be leveraged to re-analyse trial $T_{1}$ .

We characterise the predictive prior per dose $d_{\ell j}\in D_{\ell }$ by three interval probabilities; specifically, probabilities that a patient may be (i) underdosed, said to occur if the DLT risk is less than 0.16, (ii) properly dosed, if the DLT risk fall within the target interval [0.16, 0.33) and (iii) overdosed, if the DLT risk is greater than 0.33.^24^ In this data example, we suggest choosing 0.1 mg/kg to be the safe starting dose for the first-in-man trial $T_{1}$ , given $P(p_{11}<0.16|Y_{1},\ldots ,Y_{5})=0.872$ , where $Y_{1},\ldots ,Y_{5}$ denote the five hypothetical animal datasets collected from the rat and monkey studies. The choice of safe starting dose for trial $T_{2}$ will be based on both the animal data and the human toxicity data from region $R_{1}$ with a similar approach.

It will be helpful to assess the effective sample size (ESS)^15^ of the predictive priors for each $p_{\ell j},\;j=1,\ldots ,J_{\ell }$ . Before the conduct of human trials $T_{1}$ and $T_{2}$ , we approximate each marginal predictive prior for the DLT risk per dose by a Beta(*a*, *b*) distribution, for the convenience of calculating the ESS as (a+b) . The parameters *a* and *b* are determined by matching the first two moments of a Beta(*a*, *b*) with the original marginal predictive priors, obtained based on animal data. Table S1 of the Web-based Supplementary Materials reports the computed ESSs. Basically, the animal data are equivalent to what would be acquired from 4.5 to 8.2 human subjects treated in each trial.

### 3.2 Design and conduct of the phase I trials in different patient subgroups

Suppose that the phase I trials $T_{1}$ and $T_{2}$ are planned to have equal, maximum sample size, say, 24 patients. We begin by recruiting patients in cohorts of size three to trial $T_{1}$ . After the toxicity responses have been observed from the last cohort of trial $T_{1}$ , trial $T_{2}$ begins with the same trial structure. Specifically, the co-data for trial $T_{2}$ are from both the animal studies and trial $T_{1}$ . We use $h_{\ell }$ to index the cohort number of trial $T_{\ell }$ ; so $Y_{T_{1}}^{\left(h_{\ell }\right)}$ or $Y_{T_{2}}^{\left(h_{\ell }\right)}$ denote the human toxicity data accrued in cohorts $1,\ldots ,h_{\ell }$ of trial $T_{1}$ or $T_{2}$ , respectively.

Recall that we have estimated dose 0.1 mg/kg as a suitable starting dose for patients in cohort $h_{\ell }=1$ of trial $T_{1}$ . For the subsequent patient cohorts, a dose will be recommended according to the criterion

(7) $${\hat{d}}_{T_{1}}^{\left(h_{1}\right)}=\max\{d_{1j}\in D_{1}:P(p_{1j}\geq 0.33|Y_{1},\ldots ,Y_{5},Y_{T_{1}}^{\left(h_{1}-1\right)})\leq 0.25\}\;\text{for }h_{1}\geq 2$$

Phase I trials will be terminated either after completion of treatment for all 24 patients, or for safety if for any dose (including the lowest dose) the posterior risk of overdosing is too high. When the complete data from trial $T_{1}$ , denoted by $Y_{T_{1}}$ , are available, we start trial $T_{2}$ with dose

$${\hat{d}}_{T_{2}}^{\left(h_{2}=1\right)}=\max\{d_{2j}\in D_{2}:P(p_{2j}<0.16|Y_{1},\ldots ,Y_{5},Y_{T_{1}})>0.85\}$$

and dose

(8) $${\hat{d}}_{T_{2}}^{\left(h_{2}\right)}=\max\{d_{2j}\in D_{2}:P(p_{2j}\geq 0.33|Y_{1},\ldots ,Y_{5},Y_{T_{1}},Y_{T_{2}}^{\left(h_{2}-1\right)})\leq 0.25\}\;\text{for }h_{2}\geq 2$$

to be recommended to patients in the subsequent cohorts. To prevent escalating doses too rapidly, additional constraints such as ‘never skipping a dose during escalation’ may be applied in practice. This means, in our illustrative example, one cannot skip dose 0.5 mg/kg to recommend 1 mg/kg for patients in cohort 2 of trial $T_{1}$ , even if the first three doses all comply with criteria (7) and (8).

**Figure 3.**
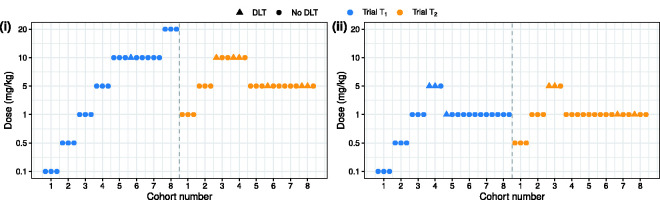
Trial trajectory of hypothetical phase I trials performed in two geographic regions, in which trial data were simulated from (i) a divergent scenario and (ii) a consistent scenario, respectively.

Figure 3 shows two simulated realisations of trials $T_{1}$ and $T_{2}$ . These data examples were simulated under different scenarios for the human dose–toxicity relationship. Subfigure (i) considers a scenario of divergent dose–toxicity relationships in regions $R_{1}$ and $R_{2}$ , while subfigure (ii) assumes the two relationships are consistent. Figure 3 verifies that the choice of a safe starting dose in trial $T_{2}$ relies on the toxicity data from trial $T_{1}$ . Reading Figure 3(i) together with Figure 2, there seems to be no relevant animal data for the first-in-man trial $T_{1}$ in scenario (i); moreover, considerable heterogeneity exists between trials $T_{1}$ and $T_{2}$ . Despite this, the proposed Bayesian approach allows the irrelevant external data to be discounted quickly in trials $T_{1}$ and $T_{2}$ , leading to declaration of doses 20 mg/kg and 5 mg/kg as the region-specific MTDs.

**Figure 4.**
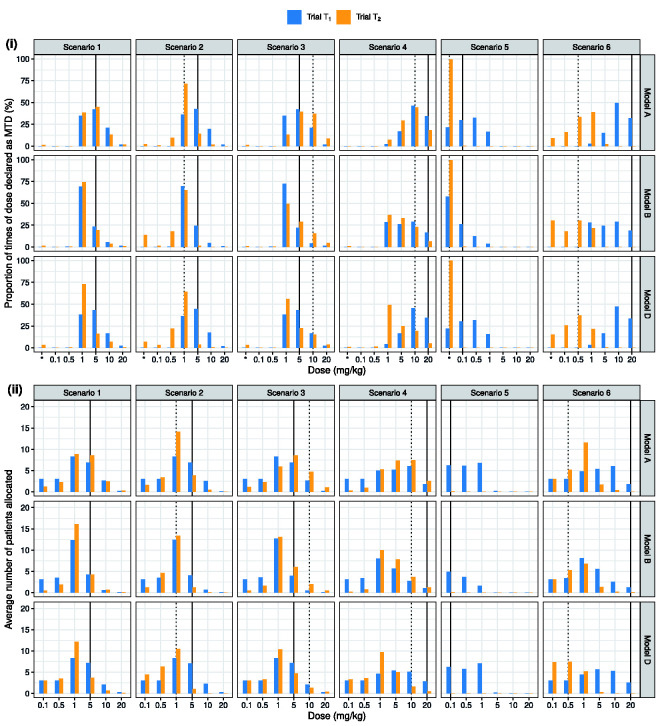
Operating characteristics of the adaptive phase I dose-escalation trials in regions $R_{1}$ and $R_{2}$ , conducted and analysed using Models A, B and D. The vertical black solid (dotted) line indicates the true MTD in the phase I trial $T_{1}$ (trial $T_{2}$ ) in each simulation scenario.

On the completion of trial $T_{1}$ , we can evaluate the posterior ESSs of the human DLT risks before the start of trial $T_{2}$ . Table 1 lists the ESSs of the marginal posteriors for the DLT risks, or say, the MAP priors for $p_{21},\ldots ,p_{2J_{2}}$ , given data from trial $T_{1}$ in scenarios (i) and (ii). As we observe, even when there exists fairly rich data from region $R_{1}$ , the MAP priors for $p_{21},\ldots ,p_{2J_{2}}$ are unlikely to dominate the estimation of MTD specific to region $R_{2}$ .

**Table 1.** Effective sample sizes of the marginal predictive posteriors (priors) for the DLT risk per dose, on the completion of trial $T_{1}$ (start of trial $T_{2}$ ), given the $T_{1}$ trial data simulated from (i) a divergent scenario and (ii) a consistent scenario, respectively

	Trial $T_{1}$						Trial $T_{2}$					
	*d*_11_	*d*_12_	*d*_13_	*d*_14_	*d*_15_	*d*_16_	*d*_21_	*d*_22_	*d*_23_	*d*_24_	*d*_25_	*d*_26_
	0.1	0.5	1	5	10	20	0.1	0.5	1	5	10	20
Sc (i)												
Posterior/Prior means	0.027	0.045	0.058	0.107	0.144	0.199	0.070	0.114	0.143	0.271	0.357	0.434
Posterior/Prior std dev.	0.031	0.042	0.048	0.068	0.079	0.105	0.110	0.141	0.158	0.222	0.260	0.280
ESS	26.3	23.4	22.7	19.7	18.8	13.5	4.3	4.1	4.0	3.0	2.4	2.1
*a*	0.7	1.1	1.3	2.1	2.7	2.7	0.3	0.5	0.6	0.8	0.9	0.9
*b*	25.6	22.3	21.4	17.6	16.1	10.8	4.0	3.6	3.4	2.2	1.5	1.2
Sc (ii)												
Posterior/Prior means	0.046	0.090	0.126	0.316	0.420	0.510	0.073	0.121	0.154	0.298	0.395	0.483
Posterior/Prior std dev.	0.043	0.058	0.064	0.156	0.210	0.233	0.111	0.138	0.153	0.206	0.242	0.259
ESS	23.0	23.4	25.8	8.0	4.7	3.7	4.5	4.6	4.6	4.0	3.1	2.7
*a*	1.1	2.1	3.3	2.5	2.0	1.9	0.3	0.6	0.7	1.2	1.2	1.3
*b*	21.9	21.3	22.5	5.5	2.7	1.8	4.2	4.0	3.9	2.8	1.9	1.4

## 4 Simulation study

In this section, we compare the operating characteristics of phase I dose-escalation trials, conducted using the proposed Bayesian hierarchical model or an alternative. The analysis models we consider are as follows:

- Model A is the proposed Bayesian model leveraging co-data from multiple sources;
- Model B discards animal data and assumes human parameter vectors $\gamma_{1}$ and $\gamma_{2}$ fully exchangeable; specifically, $w_{\ell \text{Rat}}=w_{\ell \text{Monkey}}=0,\;w_{\ell H}=1$ and $w_{\ell R}=0$ ;
- Model C analyses trials $T_{1}$ and $T_{2}$ separately, without leveraging any animal data; specifically, $w_{\ell \text{Rat}}=w_{\ell \text{Monkey}}=w_{\ell H}=0$ and $w_{\ell R}=1$ ;
- Model D leverages animal data for trials $T_{1}$ and $T_{2}$ but permits no borrowing across human subgroups; specifically, $w_{\ell \text{Rat}}=0.2,\;w_{\ell \text{Monkey}}=0.6,\;w_{\ell H}=0$ and $w_{\ell R}=0.2$ ;
- Model E analyses trial $T_{1}$ without using any co-data, and trial $T_{2}$ pooling data only from $T_{1}$ .

The prior specifications for Model A remain unchanged from Section 3.1. All the simulated $T_{1}$ trials, regardless of the analysis model, begin with the lowest dose 0.1 mg/kg. Simulated $T_{2}$ trials begin with dose 0.1 mg/kg, when implementing Models C and D. However, the choice of a safe starting dose for $T_{2}$ is conditional on both animal data and data from $T_{1}$ trial data when using analysis Model A, and solely on $T_{1}$ trial data when using Model B or E. In these settings, we select as the starting dose for trial $T_{2}$ the highest dose $d_{2j^{⋆}}$ that complies with $P(p_{2j^{⋆}}<0.16|Y_{1},\ldots ,Y_{5},Y_{T_{1}})>0.85$ for Model A, or $P(p_{2j^{⋆}}<0.16|Y_{T_{1}})>0.85$ for Model B or E. Model D is essentially to apply the model of Zheng et al.^21^ to trials $T_{1}$ and $T_{2}$ , respectively. We note that Model A simplifies to Model D if setting $w_{\ell H}=0$ while the other prior probabilities of exchangeability and non-exchangeability the same across subgroups ℓ.

Each simulated phase I trial is performed in an adaptive manner: interim dose recommendations are made according to criteria (7) and (8) for trials $T_{1}$ and $T_{2}$ , respectively. Given the true probability of toxicity per human dose listed in Table 2, we simulate human DLT outcomes from a binary distribution. We evaluate the operating characteristics under the six scenarios, comprising cases where there are conflicts across data sources, and cases where parameters in different subgroups are exchangeable. Scenarios 1 and 6 represent two extremes. Only simulated trials where all 24 patients are treated and their toxicity outcomes observed will lead to a declaration of a region-specific MTD. At the end of a completed trial in region $R_{\ell }$ , we declare the MTD as the dose satisfying

$${\hat{d}}_{\ell \text{M}}=\arg\underset{d_{\ell j}\in D_{\ell }\prime }{\min}|{\tilde{p}}_{\ell j}-0.25|$$

where ${\tilde{p}}_{\ell j}$ denotes the posterior median DLT risk at dose $d_{\ell j}$ , and $D_{\ell }\prime \subseteq D_{\ell }$ contains all the doses that were used to treat patients in trial $T_{\ell }$ , and satisfy our overdose criterion. Simulations were run in R (version 3.4.4)^30^ using the package R2OpenBUGS^31^ based on two parallel chains, each contributing 15,000 MCMC samples and sacrificing the first 5000 iterations as burn-in.

**Table 2.** Simulation scenarios for the true probability of toxicity in humans for the phase I trials $T_{1}$ and $T_{2}$ . The figure in bold indicates the target dose closest to the true MTD in each region.

	Trial $T_{1}$						Trial $T_{2}$					
	*d*_11_	*d*_12_	*d*_13_	*d*_14_	*d*_15_	*d*_16_	*d*_21_	*d*_22_	*d*_23_	*d*_24_	*d*_25_	*d*_26_
	0.1	0.5	1	5	10	20	0.1	0.5	1	5	10	20
Scenario 1	0.01	0.03	0.10	**0.25**	0.34	0.47	0.01	0.03	0.10	**0.25**	0.34	0.47
Scenario 2	0.01	0.03	0.10	**0.25**	0.34	0.47	0.05	0.12	**0.25**	0.37	0.50	0.60
Scenario 3	0.01	0.03	0.10	**0.25**	0.34	0.47	0.01	0.03	0.07	0.15	**0.25**	0.37
Scenario 4	0.01	0.03	0.05	0.08	0.15	**0.25**	0.02	0.05	0.07	0.12	**0.25**	0.36
Scenario 5	**0.25**	0.34	0.47	0.55	0.65	0.75	0.40	0.50	0.60	0.70	0.80	0.90
Scenario 6	0.01	0.03	0.05	0.08	0.15	**0.25**	0.10	**0.25**	0.36	0.50	0.60	0.68

For each toxicity scenario, we simulated 1000 pairs of adaptive phase I dose-escalation trials in regions $R_{1}$ and $R_{2}$ . In what follows, results are summarised by region. Averaging across the simulated phase I trials, we report the percentage of trials that were stopped early for safety, and percentage of trials that declared a region-specific MTD. In addition, we reported the average number of patients allocated to each dose.

Complete results from the simulation study can be found in Table S2 of the Supplementary Materials, and comparisons between Models A, C and E are presented in Figure S3. Here, we focus on comparing the operating characteristics of trials driven by Models A, B and D shown in Figure 4. We see that Model A outperforms the alternative analysis models across nearly all the simulation scenarios. In scenarios 1 and 2, where animal data are highly predictive of human DLT risks, Models A and D which leverage animal data lead to a higher percentage of correct selection (PCS) and a higher proportion of patients allocated to tolerable doses with DLT risks in the range [0.16, 0.33) than Model B. Comparing Models A and D, allowing for information sharing across patient subgroups leads to an increase in the PCS in trial $T_{2}$ from 16% to 45% in scenario 1, where DLT risks are identical across regions. Borrowing across human subgroups offers smaller but still meaningful gains in PCS in scenarios 2 and 3, when DLT risks are similar, but not identical, in regions $R_{1}$ and $R_{2}$ . Due to the ‘no-skipping-of-dose’ restriction and a small sample size, it is challenging in scenario 4 to declare doses 20 and 10 mg/kg as region-specific MTDs in trials $T_{1}$ and $T_{2}$ , respectively. Nevertheless, trials performed using Model A have the highest PCS in regions $R_{1}$ and $R_{2}$ . In particular, comparing Model A with D in scenario 4, we see an increase of 25.3% in PCS and, on average, about six more patients treated at the true MTD in trial $T_{2}$ .

In scenario 5, all the Bayesian analysis models (A–E) limit the exposure of patients to overly toxic doses, say, doses with a DLT risk exceeding 50%. Due to the use of animal data, Models A and D tend to treat more patients than Model B with doses 0.5 and 1 mg/kg, which have human DLT risks exceeding 33%. However, the average number of patients experiencing a DLT is not substantially higher than the number under Model B. Scenario 6 represents the case where the bridging assumption is incorrect. Comparing trial operating characteristics under Model A with those under Models C and D, we find trials driven by Model A allocate 6–7 more patients to dose 1 mg/kg in trial $T_{2}$ and more often incorrectly select this dose as the MTD for $R_{2}$ . However, the PCS in region $R_{2}$ remains comparable across analysis models (33.3% for Model A and 37.2% for Model D).

In scenarios 1–3, Model B assigned more patients in trial $T_{1}$ to dose 1 mg/kg than the true MTD 5 mg/kg, due to the stated ‘no-skipping’ dose-escalation constraint and our rule for defining the MTD: only administered doses are eligible to be selected as a MTD. Consequently, more trials concluded selecting a safer dose as the MTD. In scenarios 3 and 4, Model B experienced increasing difficulty distinguishing between region-specific MTDs, particularly in trial $T_{2}$ when the true MTD lies towards the top end of $D_{2}$ and when differences between human risks in different regions are relatively small. Indeed, the assumption of full exchangeability led to excessive sharing of information between the two phase I clinical trials. In scenario 5, trials in $R_{1}$ conducted using Model B were more likely to be stopped early for safety. Models A and B gave divergent operating characteristics in scenario 6. As Model B tends to underestimate the toxicity in region $R_{1}$ in such a scenario, excessive borrowing of information across regions led to more trials in $R_{2}$ stopped early than under Models A and D.

Referring to the Supplementary Materials, we can draw comparisons between the operating characteristics of dose-escalation procedures driven by Model A versus Models C and E. Models C and E can be regarded as extremes, with either permit no borrowing at all or complete pooling of human data across regions. The improved operating characteristics when comparing Models A and C should be interpreted as a mixture of the benefit from using both animal data and an appropriate bridging strategy. We have also compared Models A–E with respect to the posterior median estimates of the human DLT risks in each region, dose–toxicity relationship in each human subgroup. Figure S4 of the Supplementary Materials show that Model A outperforms the others, providing very satisfactory characterisation of the association on the termination of a phase I clinical trial. We additionally ran simulations for a robust version of Model B with $w_{\ell R}=0.20$ . Conclusions are similar with those written in Neuenschwander et al.^16^ and Zheng et al.^21^ on the advantage of including a robust weakly informative distribution, and thus will not be repeated in this paper.

**Figure 5.**
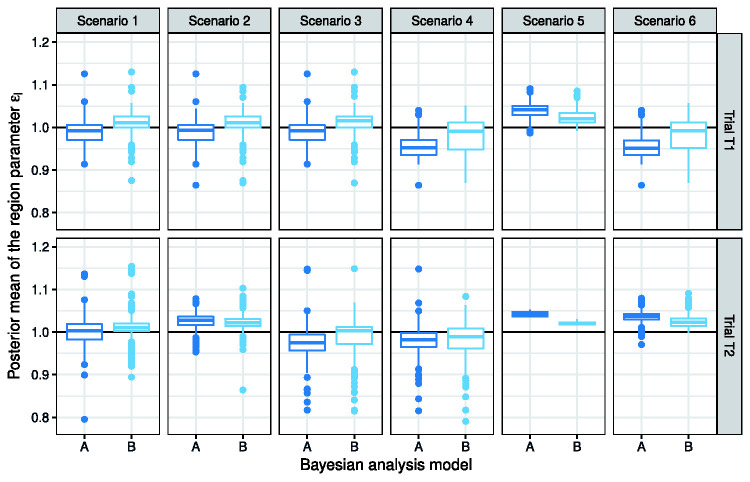
Boxplots that depict the posterior means of the region parameter $\epsilon_{\ell }$ estimated by the end of completed trials, designed using Model A or Model B. The horizontal black line represents the prior mean of $\epsilon_{\ell }$ .

We introduced bridging parameters $\epsilon_{1},\;\epsilon_{2}$ into the human dose–toxicity models. Model A maps animal data onto the equivalent human dosing scale for the incorporation into human trials $T_{1}$ and $T_{2}$ . With inclusion of a random $\epsilon_{\ell }$ after setting $\delta_{A_{i}}=1$ for the human trials, the posterior distribution of the bridging parameters captures whether animal data over- or under-predicted the DLT risk in a specific human subgroup. See Figure 5 for the boxplots of Model A in scenario 4, where rat and monkey data lead to under-estimation of human DLT risks. We observe the posterior means of *ϵ*_1_ and *ϵ*_2_ are shifted downwards from the prior mean of 1. When the posterior mean of $\epsilon_{\ell }$ is shifted to take a value larger than 1, it suggests animal data are likely to have overestimated the human DLT risks; see, for example, scenario 2 for trial $T_{2}$ . In scenario 5, many simulated trials are stopped early for safety, and since $\epsilon_{\ell }$ is only estimated for a completed trial, we exclude results for this scenario in Figure 5. In contrast, the parameter $\epsilon_{\ell }$ embedded in Model B exclusively addresses the intrinsic differences arising from ethnicity between patient subgroups. Within the same scenario, say, scenarios 1 and 3 where the bridging assumption is correct, *ϵ*_1_ and *ϵ*_2_ take values centring around 1 (the normal prior mean). When the drug is more toxic in region $R_{1}$ than $R_{2}$ , the posterior means of *ϵ*_2_ tend to be larger; for example, see results for scenarios 2 and 6.

Additional simulations were performed to support future application of the proposed Bayesian hierarchical model. In particular, we evaluated the impact of prior probabilities of exchangeability and non-exchangeability, i.e. $w_{\ell S_{1}},\ldots ,w_{\ell S_{K}},w_{\ell R}$ , for ℓ=1,2 (Section C of the Web-based Supplementary Materials). We find that a large $w_{\ell S_{k}}$ means the MAP prior would likely be dominated by the corresponding BVN $(\mu_{S_{k}},\Psi ),\;k=1,\ldots ,K$ , and therefore could lead to much enhanced operating characteristics in scenarios of prior-data consistency. Whereas, this is at the cost of increased difficulty for down-weighting any animal data in scenarios of prior-data inconsistency. Additionally, we compare the proposed methods to the bridging CRM in Liu et al.^12^ (Section D of the Supplementary Materials). As expected, the inclusion of animal data in the escalation procedure yields improved performance when such information is relevant.

## 5 Discussion

Bridging studies have received considerable interest,^32^ as fewer resources may be needed to demonstrate drug behaviours by using relevant data from other subgroups, compared with the approach of establishing an independent, complete package of clinical drug development. Statistical methodology to extrapolate across geographic regions has been proposed mainly in the context of phase II and phase III clinical trials.^33–36^ Much less has been written on phase I clinical trials, where different metrics are used to evaluate trial efficiency and estimation accuracy.

In this paper, we seek to improve decision making in a phase I bridging study, by leveraging not only the trial data on an original subgroup/region for drug registration, but also preclinical animal data. The novelty of the proposed methodology is relating to sensible constellations of parameter vectors. Technically speaking, the hierarchy of the proposed model is constructed by placing the human dose–toxicity parameter vectors $\gamma_{1},\ldots ,\gamma_{L}$ at the same level as the standardised, study-specific, animal parameter vectors $\theta_{1},\ldots ,\theta_{M}$ ; for local robust inferences about each $\gamma_{\ell },\;\ell =1,\ldots ,L$ , we split the full probability of exchangeability into fractions $w_{\ell S_{1}},\ldots ,w_{\ell S_{K}},w_{\ell H}$ and $w_{\ell R}$ , which sum up to 1. Moreover, independent weakly-informative priors BVN $\left(m_{0\ell },R_{0\ell }\right)$ are placed on $\gamma_{1},\ldots ,\gamma_{L}$ , respectively, for the possibility of non-exchangeability. Gains in operating characteristics can therefore be attributed to the incorporation of consistent animal data or data from the original geographic region, or both.

In our simulation study, we use the prior probabilities $w_{\ell S_{1}},\ldots ,w_{\ell S_{K}},w_{\ell H}$ and $w_{\ell R}$ that have been specified individually for subgroups ℓ=1,2 . Nonetheless, it is viable to tightly associate the prior probabilities for the bridging trial $T_{2}$ with the posterior probabilities obtained by the end of the trial $T_{1}$ . Such specification has pros and cons. As noted by one anonymous reviewer, this could facilitate the understanding towards (dis)similarity between toxicity profiles in animals and humans. An unfavourable scenario, however, could easily be envisaged. That is, the maximally attainable operating characteristics could be limited, when the toxicity in humans is very distinct across regions $R_{1}$ and $R_{2}$ . Future practitioners may choose these prior probabilities on a case-by-case basis, although extensive simulations presented in this paper have assured a robust inference about the region-specific MTDs.

We note that the proposed methodology has wider applications. For example, there may be a need to design phase I dose-escalation trials in subgroups defined by clinical or genetical characteristics which could potentially modify the therapeutic effect of the drug.^37^ Based on our Bayesian model, information from patient subgroups with similar safety profiles can be leveraged. There is no restriction on the number of studies that will be run in the new patient subgroups, nor on the number of subgroups to provide the co-data. When a large number of subgroups are involved, estimate of parameters that represent the between-trial heterogeneity (specifically, $\tau_{3},\;\tau_{4}$ ) tends to be more accurate. It would therefore better determine the degree of borrowing across human trials $T_{1},\ldots ,T_{L}$ .

This paper has focused on the design of bridging studies to estimate the MTD in a new geographic region or human subgroup of a previously studied disease indication, although the approach can also be used for other settings. Future work could consider extending the proposed hierarchical model to accommodate the case of bridging across subgroups in related disease indications, when distinct endpoints, different dosing schedules or formulations, etc. might be necessary. The research question is highly relevant within the paradigm for precision medicine. A new class of efficient approaches, known as basket trials,^38^ have emerged, where the same treatment is tested in potentially heterogeneous patient subgroups (often defined by genetic characteristics). Robust hierarchical models have been considered for borrowing of information.^39,40^ The proposed methodology can potentially be used to analyse phase I oncology basket trials, where multiple cancer subtypes are studied under a *master protocol.*^41^ It is conceptually similar to the proposal by Neuenschwander et al.^39^: prior probabilities of exchangeability and non-exchangeability are assigned independently to each vector of subgroup-specific dose–toxicity model parameters. Our Bayesian model allows co-data to contribute towards formulating the exchangeability distributions so as to discuss borrowing of information from specific sources. Independent non-exchangeability distributions ensure we obtain the robust estimates of the dose–toxicity model parameters underpinning extreme subgroups. Improving statistical inferences for extreme subgroups, which could be similar amongst themselves, is outside of the scope of the present research. This is an area for future research, with related investigation undertaken in the context of phase II basket trials to enable information sharing based on distributional discrepancy between model parameters for therapeutic effects.^42^

## Supplemental Material

sj-pdf-1-smm-10.1177_0962280220986580 - Supplemental material for Bridging across patient subgroups in phase I oncology trials that incorporate animal data Supplemental material, sj-pdf-1-smm-10.1177_0962280220986580 for Bridging across patient subgroups in phase I oncology trials that incorporate animal data by Haiyan Zheng, Lisa V Hampson and Thomas Jaki in Statistical Methods in Medical Research
