# Supplementary material for: Bridging across patient subgroups in phase I oncology trials that incorporate animal data
Source: Stat Methods Med Res. 2021 Jan 27;30(4):1057–71. doi: 10.1177/0962280220986580 (PMC8129464; doi:10.1177/0962280220986580)
Supplement: sj-pdf-1-smm-10.1177_0962280220986580 - Supplemental material for Bridging across patient subgroups in phase I oncology trials that incorporate animal data [file sj-pdf-1-smm-10.1177_0962280220986580.pdf]

Web-based Supplementary Materials for: Bridging across patient subgroups in phase I oncology trials that incorporate animal data

by Haiyan Zheng, Lisa V. Hampson, Thomas Jaki

A. THE SIMULATED ANIMAL DATA AND PREDICTIVE PRIORS FOR HUMAN TOXICITY

Figure S1 represents the simulated animal data used for the numerical studies. The height of the bar represents the number of animal subjects treated, and the height of the dark grey segment counts the number of toxicity. Doses listed in brown are those administered to either rats and monkeys, which are translated onto an equivalent human dosing scale in black. Projections are made by scaling animal doses using the prior median of δ_{Rat} or δ_{Monkey}.

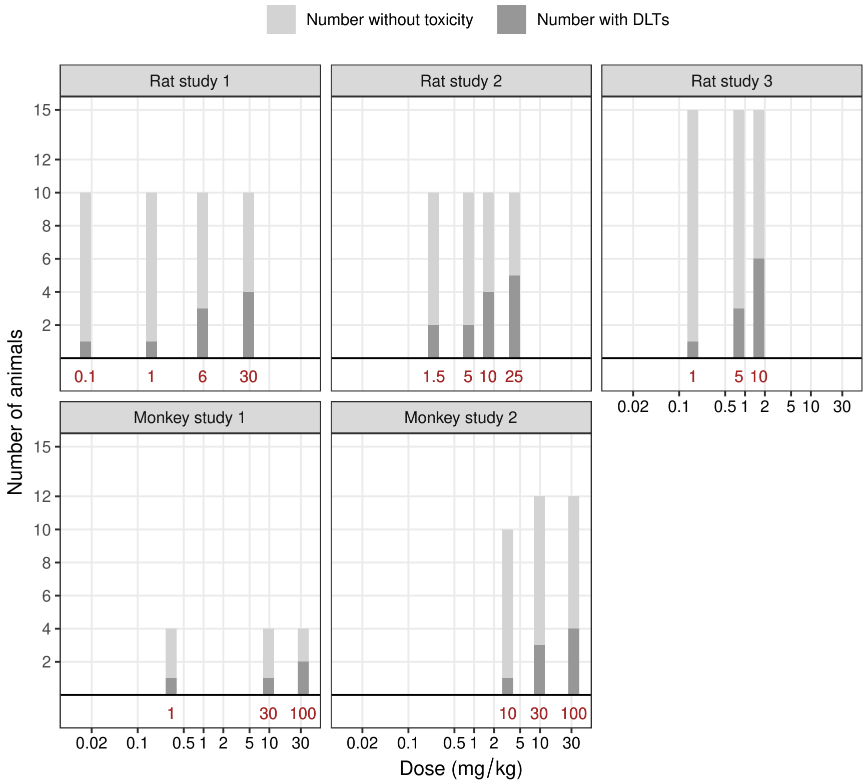

Figure S1: Hypothetical preclinical data in rats and monkeys, simulated for the illustrative examples as well as the simulation study.

With the simulated animal data and the priors specified in Section 3.1, we further obtain the marginal predictive priors that are shown graphically in Figure S2. We approximate these marginal predictive priors with a series of Beta(a, b) distributions and report the prior effective sample size (ESS) for the predicted human toxicity at each dose in Table S1.

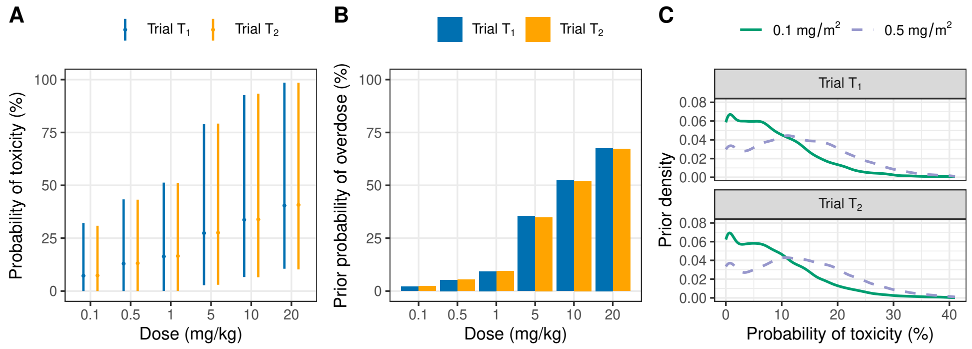

Figure S2: Summaries about the robust, marginal predictive priors for human toxicity based on the animal data. Panel A shows median and 95% credible interval of the marginal predictive prior for human toxicity at each dose. Panel B presents the prior interval probability of overdose, and Panel C displays prior densities for the risks of toxicity at potential starting doses.

Table S1: Effective sample sizes of the marginal predictive priors for the DLT risk per dose, obtained before trial $\mathcal{T}_1$ begins by fitting the robust Bayesian model, which translates the animal data onto an equivalent human dosing scale.

	Trial $\mathcal{T}_1$						Trial $\mathcal{T}_2$					
	d_{11} 0.1	d_{12} 0.5	d_{13} 1	d_{14} 5	d_{15} 10	d_{16} 20	d_{21} 0.1	d_{22} 0.5	d_{23} 1	d_{24} 5	d_{25} 10	d_{26} 20
Prior means	0.095	0.148	0.181	0.302	0.376	0.446	0.094	0.148	0.182	0.304	0.378	0.449
Prior std dev.	0.101	0.122	0.134	0.173	0.199	0.216	0.095	0.118	0.131	0.174	0.200	0.217
ESS	7.4	7.5	7.3	6.0	4.9	4.3	8.4	8.1	7.7	6.0	4.9	4.3
a	0.7	1.1	1.3	1.8	1.9	1.9	0.8	1.2	1.4	1.8	1.8	1.9
b	6.7	6.4	5.9	4.2	3.1	2.4	7.6	6.9	6.3	4.2	3.0	2.3

B. SIMULATION RESULTS THAT ARE NOT PRESENTED IN THE MAIN PAPER

In the main mauscript, we have compared Model A (the proposed approach) with Models B and D and interpreted the results in terms of benefit attributed to either using the robust bridging strategy (Model A versus Model B) or animal data (comparing Model A and D). Here, we present additional simulation results in Figure S3 to compare our Model A with Models C and E, which does not use co-data at all and which completely pools the data from trial $\mathcal{T}_1$ to $\mathcal{T}_2$, respectively. Numerical results of the complete comparison based on Models A – E are listed in Table S2.

In scenarios 1 – 3 when the bridging assumption is correct and/or animal data provide high predictability of human toxicity, it is evident that Model A leads to significantly increased PCS and allocates more patients to the true MTD in both regions $\mathcal{R}_1$ and $\mathcal{R}_2$. For example, comparing Models A and C, the PCS was increased from 14.7% to 42% in scenario 1 for trial $\mathcal{T}_1$ due to the use of animal data, and from 17% to 45% for trial $\mathcal{T}_2$ due to the use of animal data and correct bridging strategy. Model E does not leverage animal data into trial $\mathcal{T}_1$ but completely pools in the $\mathcal{T}_1$ trial data into trial $\mathcal{T}_2$. As a result, we observe that the PCS for trial $\mathcal{T}_1$ was much lower than that of Model A in these scenarios, although a higher PCS is observed in scenario 2 for pooling in the consitent $\mathcal{T}_1$ trial data. Using trial data from other human subgroup by such completely pooling approach could be harmful, which is evidenced by the results in scenario 6. Comparing Model A and Model E, we see that the latter allocated much more patients to excessively toxic doses which are 5, 10 and 20 mg/kg in this scenario, and more often decleared dose 1 mg/kg as the MTD. This is not surprising: pooling in the $\mathcal{T}_1$ trial data means we suppose patients in regions $\mathcal{R}_1$ and $\mathcal{R}_2$ are exchangeable, and Model E would consequently underestimate the human toxicity in trial $\mathcal{T}_2$.

We now switch our focus to assessing the accuracy of the posterior point estimates based on the Bayesian analysis models. As has been illustrated, leveraging co-data that are (in)consistent with current trial data, should improve the accuracy of posterior estimates for the probability of

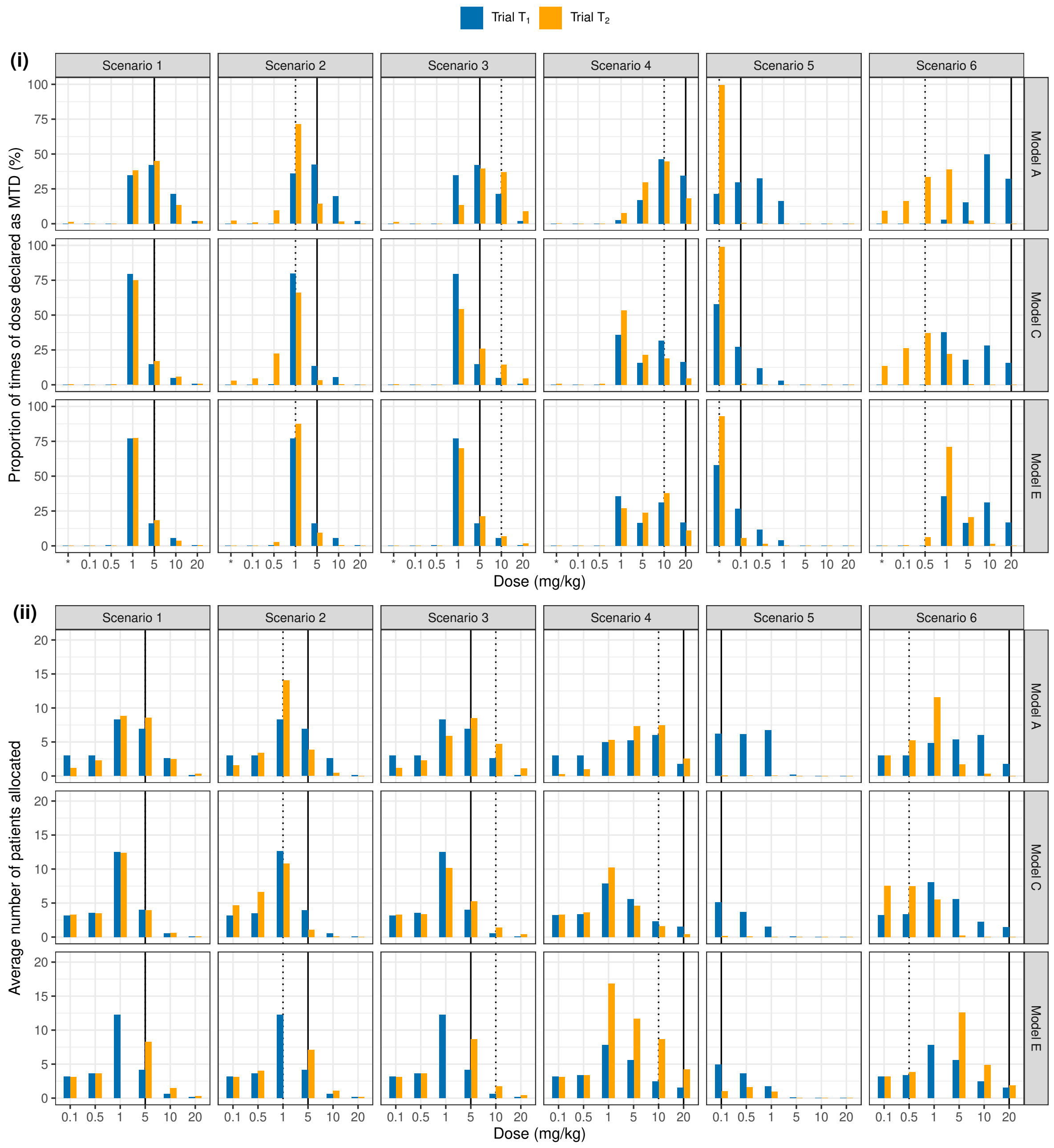

Figure S3: Operating characteristics of the adaptive phase I dose-escalation trials in two geographic regions, conducted and analysed using Models A, C, D. The vertical black solid (dotted) line indicates the true MTD in the western trial $\mathcal{T}_1$ (eastern trial $\mathcal{T}_2$) in each simulation scenario.

toxicity. Analysis models that enable borrowing of information in this situation correspondingly will present (dis)advantages over those not permitting borrowing at all. We are thus much concerned with the accuracy of posterior estimates based on the proposed Bayesian model compared with its alternatives. In the simulation study, we preserve the point estimates (posterior medians) by the end of the *completed* trials, which may be a subset of or the entire 1000 pairs. We average across such point estimates per human dose under the analysis models A – E to approximate the dose-toxicity relationship specific to each region.

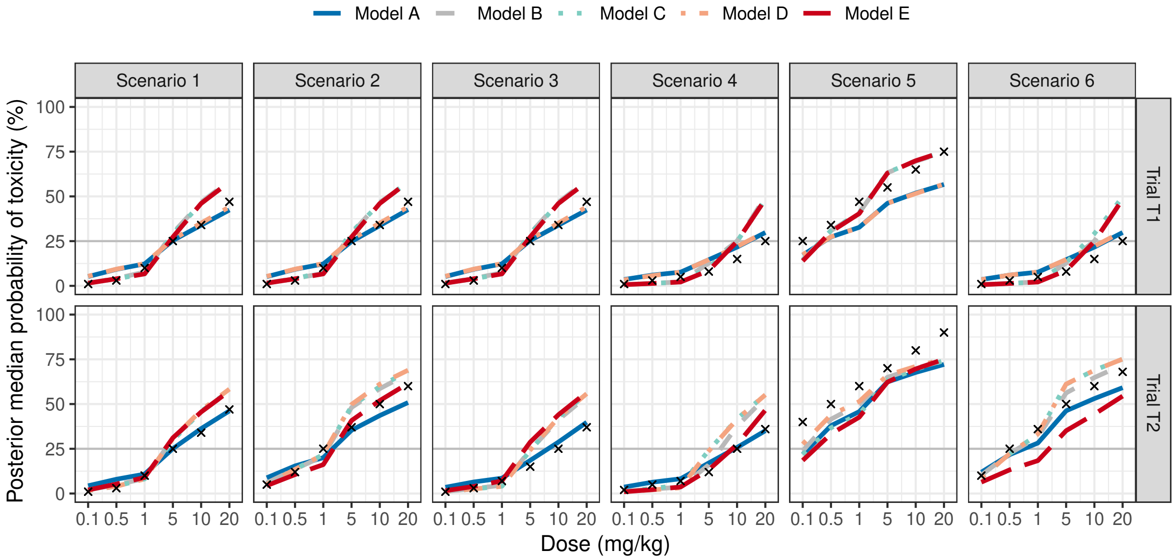

Figure S4: Average fitted dose-toxicity curves obtained under Models A – E, based on the *completed* trials only. The black cross marks the true probability of toxicity per human dose of interest for the six simulation scenarios. The horizontal gray line indicates the target level, at which the MTD is defined.

Figure S4 visualises how close the estimated dose-toxicity relationships would be to the true human toxicicty, which is marked with black cross per dose of interest. As we can see, Model C and Model E showed less satisfactory performance, compared with the rest, across nearly all the simulation scenarios. The dose-toxicity curves fitted using these two models are severely deviated from the true dose-toxicity relationship when the region-specific MTDs are very divergent, such as in scenario 6. Model B bears excessive sharing of information, which is therefore inappropriate for the divergent scenarios, either. In contrast, Models A and D give quite robust estimates about the dose-toxicity relationship in most cases. Moreover, these models converge well to the true MTD. Scenario 5 corresponds to an overly toxic situation, where most simulated trials are meant to be *stopped early*. The complement subset, i.e., *completed* trials, are those suggested the drug to be much less toxic than the fact, given fewer DLTs observed. Consequently, the fitted curves obtained from the *completed* trials only tend to underestimate the true probability of toxicity.

C. ON THE PRIOR PROBABILITIES OF (NON-)EXCHANGEABILITY

The operating characteristics of the proposed Bayesian hierarchical model for bridging studies also rely on the specification of prior probabilities of exchangeability and non-exchangeability. Of our particular interest is to investigate the impact of misspecifying such prior probabilities on the model performance in (i) estimating the MTD correctly and (ii) allocating patients to appropriate doses. For example, how the proposed procedure behaves if specifying a moderate to large prior probability of exchangeability to animal species, of which the toxicity profile actually is not commensurate with human's.

We use the two most challenging scenarios from the simulation study, i.e., scenario 4 where animal data do not predict the human toxicity well, and scenario 6 where animal data may suffice to support the estimation of MTD only in Trial $\mathcal{T}_2$ while the bridging assumption does not hold. Focusing on the proposed Bayesian hierarchical model (Model A), we simulate 1,000 pairs of the phase I oncology trials, $\mathcal{T}_1$ and $\mathcal{T}_2$ setting the prior probabilities of exchangeability and

non-exchangeability as follows.

- Wgt1:

$$w_{1\text{Rat}} = 0.2, w_{1\text{Monkey}} = 0.6,\ w_{1\mathcal{H}} = 0,\ w_{1R} = 0.2$$
$$w_{2\text{Rat}} = 0.1, w_{2\text{Monkey}} = 0.5,\ w_{2\mathcal{H}} = 0.2,\ w_{2R} = 0.2$$

- Wgt2:

$$w_{1\text{Rat}} = 0.3, w_{1\text{Monkey}} = 0.5,\ w_{1\mathcal{H}} = 0,\ w_{1R} = 0.2$$
$$w_{2\text{Rat}} = 0.2, w_{2\text{Monkey}} = 0.4,\ w_{2\mathcal{H}} = 0.2,\ w_{2R} = 0.2$$

- Wgt3:

$$w_{1\text{Rat}} = 0.4, w_{1\text{Monkey}} = 0.4,\ w_{1\mathcal{H}} = 0,\ w_{1R} = 0.2$$
$$w_{2\text{Rat}} = 0.3, w_{2\text{Monkey}} = 0.5,\ w_{2\mathcal{H}} = 0.2,\ w_{2R} = 0.2$$

- Wgt4:

$$w_{1\text{Rat}} = 0.5, w_{1\text{Monkey}} = 0.3,\ w_{1\mathcal{H}} = 0,\ w_{1R} = 0.2$$
$$w_{2\text{Rat}} = 0.4, w_{2\text{Monkey}} = 0.2,\ w_{2\mathcal{H}} = 0.2,\ w_{2R} = 0.2$$

- Wgt5:

$$w_{1\text{Rat}} = 0.6, w_{1\text{Monkey}} = 0.2,\ w_{1\mathcal{H}} = 0,\ w_{1R} = 0.2$$
$$w_{2\text{Rat}} = 0.5, w_{2\text{Monkey}} = 0.1,\ w_{2\mathcal{H}} = 0.2,\ w_{2R} = 0.2$$

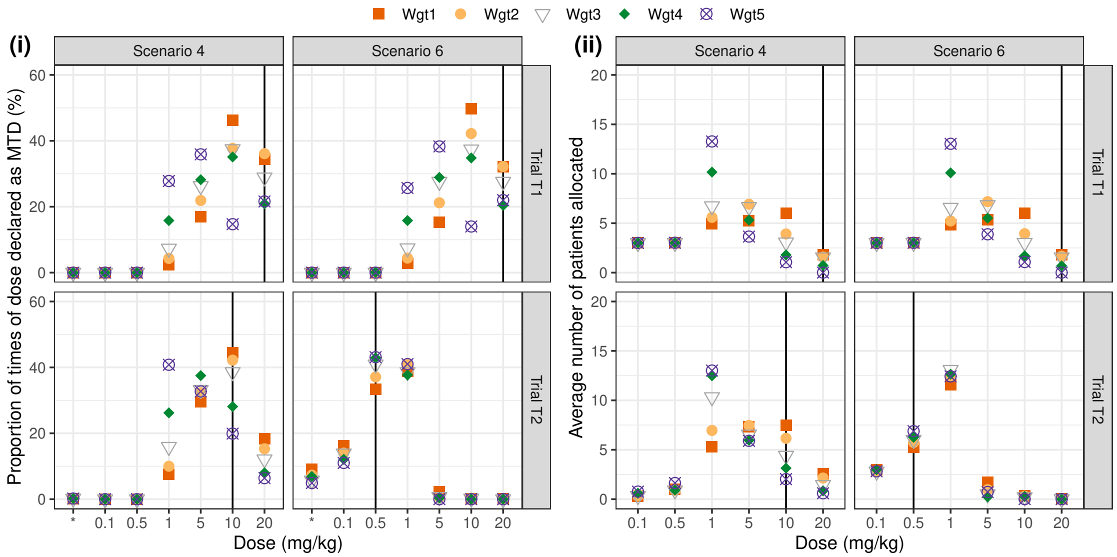

Figure S5: Operating characteristics of the adaptive phase I dose-escalation trials, conducted and analysed using Model A under various sets of prior probabilities of exchangeability and non-exchangeability. The vertical black solid (dotted) line indicates the true MTD in the western trial $\mathcal{T}_1$ (eastern trial $\mathcal{T}_2$) under in simulation scenario.

Figure S5 shows that in these two scenarios, Wgt1 and Wgt2 lead to higher proportion of times for correctly estimating the MTD. These two sets of weights set a relatively low weight on the rat data that predict dose 1 mg/kg as the most probable MTD (in fact far from the true MTD), making the dose-escalation procedure easier to down-weight such incommensurate animal data. By contrast, in $\mathcal{T}_2$ under scenario 6, the Wgt5 that set relatively high weight on the rat data presents advantages brought by the data commensurability. Looking at the subfigure (ii) for the average number of patients allocated per dose, the Wgt 1 to Wgt 3 all yield fairly even allocation while Wgt 4 and Wgt 5 concentrate patients on the low doses, particularly 1 mg/kg.

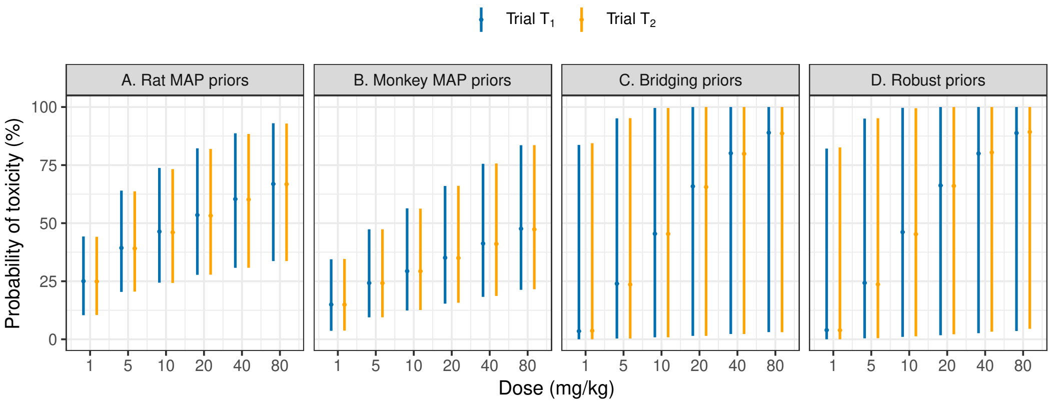

Figure S6: Summaries about the predictive priors for human toxicity, when using animal data from a single species (Panels A and B) or no animal data at all (Panels C and D). Medians together with 95% credible intervals of the marginal predictive priors are plotted.

It would be of interest to check how the proposed methodology performs when the human equivelant doses, as translated from animal data based on δ_{Rat} and δ_{Monkey}, are far from the actual doses used in the human trials. We thus consider two new simulation scenarios A and B, which are adapted from the scenarios 4 and 6 investigated above, respectively. More specifically, the doses available for evaluation in trials $\mathcal{T}_1$ and $\mathcal{T}_2$ are far larger than the highest probable human equivalent doses after the translation across species, i.e., $\mathcal{D}_\ell = \{1, 5, 10, 20, 40, 80\}$ for $\ell = 1, 2$, but the risks of toxicity remain identical to the levels specified in Table 2 of the main paper. This creates a larger discrepancy in the toxicity profile between animals and humans. Figure S6 summarises the meta-analytic predictive (MAP) priors for the risk of toxicity by source of information on the scale of human doses for evaluation. It suggests that even in this new configuration of human doses, animal data (particularly those collected from monkey studies) might still give relevant prediction towards human toxicity.

Adhering the same specification of simulation, we investigate two sets of prior probabilities of exchangeability and non-exchangeability featured by different levels of $w_{\ell R} = 0.2, 0.5$ for robust inferences:

- $w_{\ell R} = 0.2$:

$$w_{1\text{Rat}} = 0.2, w_{1\text{Monkey}} = 0.6,\ w_{1\mathcal{H}} = 0,\ w_{1R} = 0.2$$
$$w_{2\text{Rat}} = 0.1, w_{2\text{Monkey}} = 0.5,\ w_{2\mathcal{H}} = 0.2,\ w_{2R} = 0.2$$

- $w_{\ell R} = 0.5$:

$$w_{1\text{Rat}} = 0.2, w_{1\text{Monkey}} = 0.3,\ w_{1\mathcal{H}} = 0,\ w_{1R} = 0.5$$
$$w_{2\text{Rat}} = 0.1, w_{2\text{Monkey}} = 0.1,\ w_{2\mathcal{H}} = 0.3,\ w_{2R} = 0.5$$

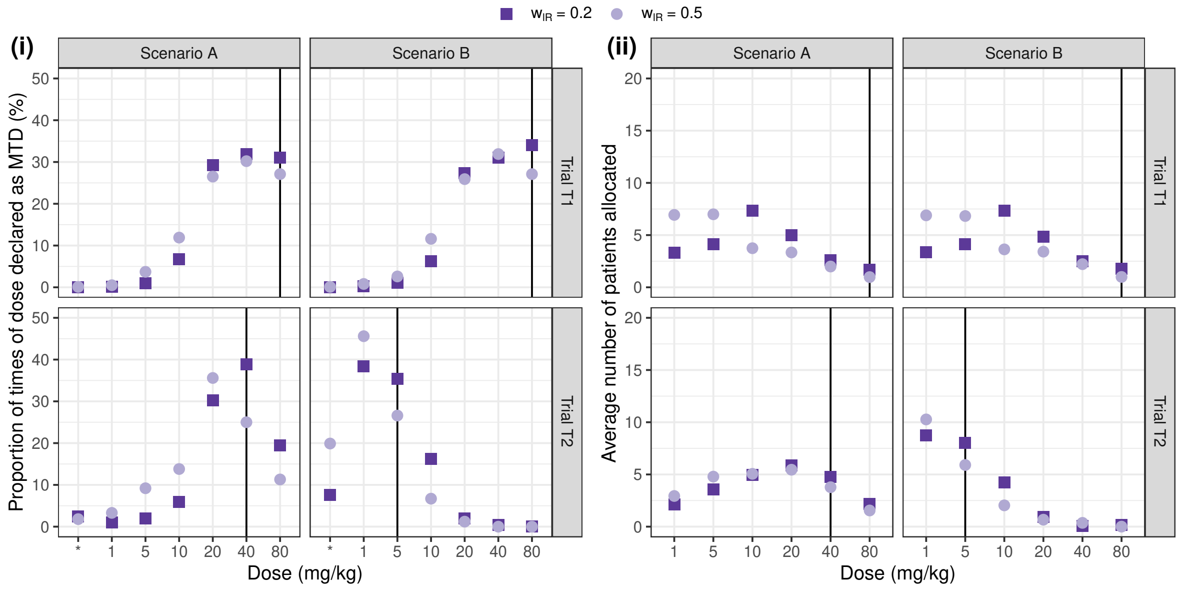

Figure S7: Operating characteristics of the adaptive phase I dose-escalation trials, conducted and analysed using Model A when different levels of prior probability of non-exchangeability are considered.

Figure S7 compares the operating characteristics of the simulated phase I trials, conducted and analysed by using different levels of $w_{\ell R}$. As we can read from subfigure (i), by increasing $w_{\ell R}$ from 0.2 to 0.5 to downplay the available animal data, the percentage of correctly selecting the MTD (marked by the black vertical line on the plots) is reduced per scenario, especially for trial $\mathcal{T}_2$, for which monkey data could be thought of as relevant. With subfigure (ii), we see that setting $w_{\ell R} = 0.2$ (overwhelming prior weight has been given to the monkey data) favours the allocation of patients to doses 5 to 20 mg/kg, which are suggested to be the probable target dose.

D. COMPARISON WITH THE BRIDGING CONTINUAL REASSESSMENT METHOD

As suggested by one anonymous reviewer, we investigate how the proposed Bayesian hierarchical model compares with the bridging continual reassessment method (CRM) by Liu et al. (2015) [3]. Although the latter was not meant to be used for incorporating animal data, it has been widely recognised in the field of phase I bridging trials, particularly in oncology. It is important to state at the outset that the aim of the following simulation study is not to compare the two Bayesian models which involve two distinct modelling strategies, but to illustrate the relative strength and weakness of each, when applied for the conduct of phase I dose-escalation trials in relevant patient subgroups.

For the present purpose, when implementing the bridging CRM, we use the medians of the robust MAP priors that synthesise both rat and monkey data, i.e., (0.095, 0.148, 0.181, 0.302, 0.376, 0.446) plotted using points in Panel A of Figure S2, as the skeleton probabilities for the CRM model to guide the dose-escalation procedure in trial $\mathcal{T}_1$. Following Liu et al. (2015) [3], three sets of skeleton probabilities are generated by the end of trial $\mathcal{T}_1$, based on the posterior medians of the toxicity risks per human dose. This leads to three CRM models, each with a probability of 1/3 for the model being true. The dose-escalation procedure in trial $\mathcal{T}_2$ is then a result of the model averaging. Different from Liu et al. (2015) [3] who select the interim recommendation doses based on the point estimate (posterior mean) of toxicity, we adopt the same criterion as specified in our main paper to recommend a maximum dose, of which the probability of overdose is controlled

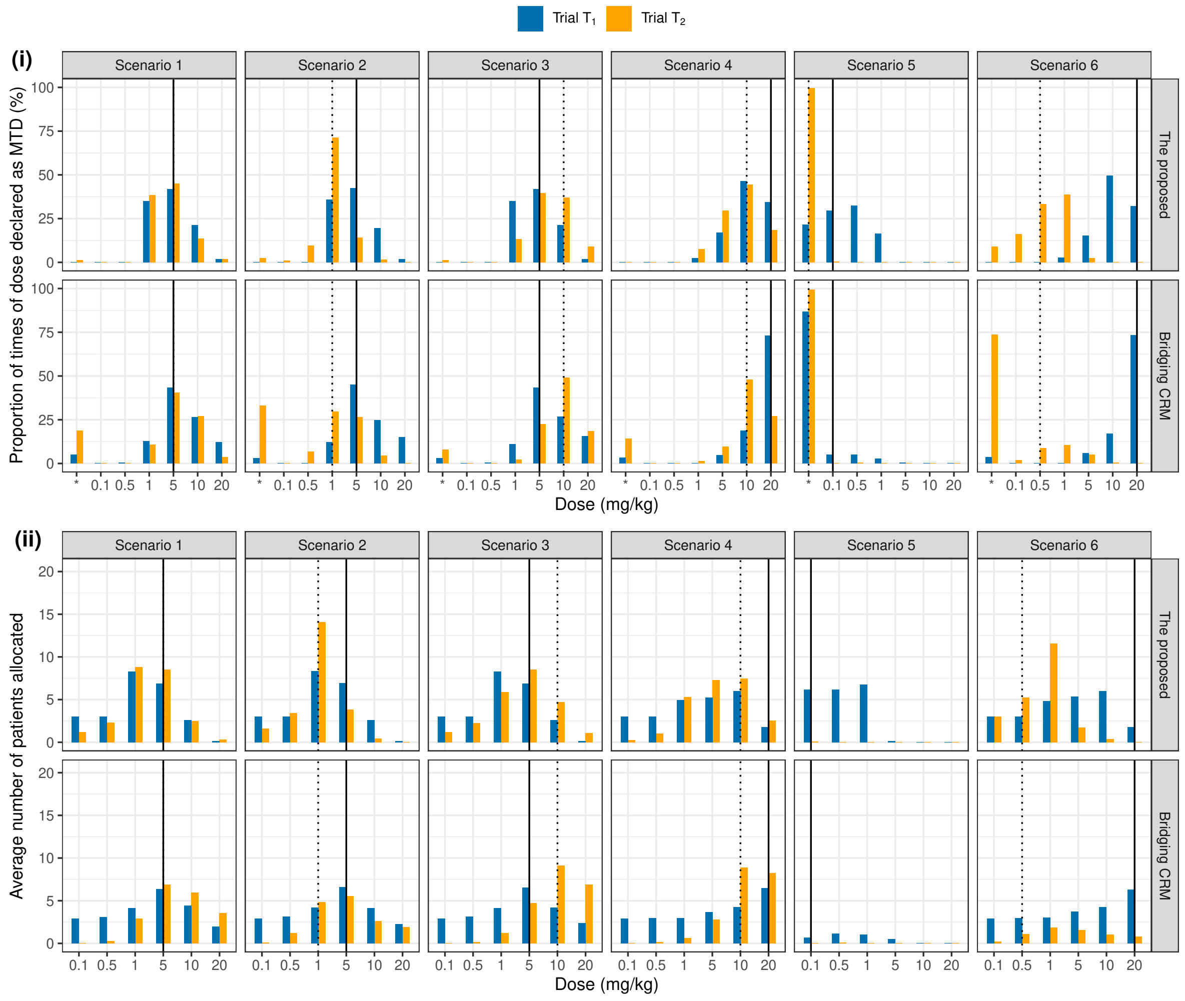

Figure S8: Comparison of the proposed Model A against the bridging CRM from an application perspective, as used to guide the adaptive phase I dose-escalation trials in regions $\mathcal{R}_1$ and $\mathcal{R}_2$.

below 25%. The way we choose a starting dose in trial $\mathcal{T}_2$, and the constraints of early stopping for safety as well as "never skipping a dose during escalation" are also kept the same to what we will use for implementing the proposed Model A.

Figure S8 presents the simulation results for the trial operating characteristics yielded by the proposed Model A against the bridging CRM. In scenarios 1 and 2 where the co-data are consistent for both trials $\mathcal{T}_1$ and $\mathcal{T}_2$, the proposed Model A is more advantageous than the bridging CRM in correctly selecting the MTD particularly in trial $\mathcal{T}_2$ as well as allocating more patients to this dose. By contrast, the bridging CRM displays better performance particularly in scenario 4 where the inconsistent co-data are weighed considerably by the proposed Model A. Combining results in scenarios 5 and 6, the bridging CRM in our implementation is more prone to early stopping, while the proposed Model A gives satisfactory operating characteristics.

As we noted, this simulation study is mainly to give an intuition about both Bayesian models when applied in phase I bridging trials. Each of them has a number of parameters that could be tuned for the best model performance possible. We recommend extensive simulations to be done for either model that would be chosen for future implementation.

Table S2: Comparison of alternative analysis models in terms of the percentage of selecting a dose as MTD at the end of the trials, percentage of early stopping for safety, average patient allocation, and average number of patients with toxicity.

	Design		% dose declared as MTD & average patient allocation															$\bar{N}_{\mathcal{T}_1}$	$\bar{N}_{\mathcal{T}_2}$
			Trial $\mathcal{T}_1$							Trial $\mathcal{T}_2$								
			d_{11} 0.1	d_{12} 0.5	d_{13} 1	d_{14} 5	d_{15} 10	d_{16} 20	None	d_{21} 0.1	d_{22} 0.5	d_{23} 1	d_{24} 5	d_{25} 10	d_{26} 20	None		
Scenario 1		pTox	0.01	0.03	0.10	**0.25**	0.34	0.47		0.01	0.03	0.10	**0.25**	0.34	0.47			
	Benchmark	Sel	0	0.3	14.6	**59.1**	22.0	4.0		0	0.3	14.6	**59.1**	22.0	4.0			
	Model A	Sel	0	0	34.9	**42.0**	21.2	1.9	0	0	0	38.4	**45.0**	13.5	1.9	1.2		
		Pts	3.0	3.0	8.3	6.9	2.6	0.2		1.2	2.3	8.8	8.5	2.5	0.3		24.0	23.6
	Model B	Sel	0	0.6	69.0	**23.6**	5.3	1.4	0.1	0	0.5	74.1	**19.2**	3.7	0.9	1.6		
		Pts	3.1	3.5	12.3	4.3	0.6	0.1		0.5	1.9	16.1	4.3	0.7	0.1		23.9	23.6
	Model C	Sel	0	0.3	79.4	**14.7**	4.8	0.7	0.1	0	0.6	75.2	**17.0**	6.0	0.7	0.5		
		Pts	3.2	3.6	12.5	4.0	0.6	0.1		3.3	3.5	12.4	4.0	0.6	0.1		24.0	23.9
	Model D	Sel	0	0.1	37.9	**43.0**	16.7	2.3	0	0	0.6	73.0	**16.0**	6.7	0.6	3.1		
		Pts	3.0	3.0	8.3	7.1	2.1	0.4		3.1	3.5	12.2	3.7	0.7	0.2		23.9	23.4
	Model E	Sel	0	0.4	77.0	**16.2**	5.7	0.6	0.1	0	0	77.3	**18.4**	3.6	0.5	0.2		
		Pts	3.1	3.6	12.3	4.1	0.7	0.1		0	0	18.9	4.1	0.7	0.2		23.9	23.9
Scenario 2		pTox	0.01	0.03	0.10	**0.25**	0.34	0.47		0.05	0.12	**0.25**	0.37	0.50	0.60			
	Benchmark	Sel	0	0.3	14.6	**59.1**	22.0	4.0		0.7	18.9	**56.8**	21.4	2.2	0			
	Model A	Sel	0	0	36.0	**42.4**	19.6	2.0	0	1.0	9.5	**71.3**	14.2	1.7	0	2.3		
		Pts	3.0	3.0	7.9	**7.7**	2.3	0.1		3.0	3.5	12.5	4.4	0.6	0		24.0	23.5
	Model B	Sel	0	0.7	69.5	**24.2**	4.6	1.0	0	1.6	17.8	**65.1**	1.5	0.2	0	13.8		
		Pts	3.1	3.5	12.5	4.1	0.7	0.1		1.3	4.7	13.4	1.2	0.1	0		24.0	20.7
	Model C	Sel	0	0.5	80.0	**13.4**	5.7	0.3	0.1	4.6	22.6	**66.2**	3.2	0.5	0	2.9		
		Pts	3.2	3.5	12.6	4.0	0.6	0.1		4.7	6.6	10.8	1.1	0.1	0		24.0	23.3
	Model D	Sel	0	0	36.1	**44.4**	17.4	2.1	0	3.1	22.0	**64.0**	3.6	0.4	0.1	6.8		
		Pts	3.0	3.0	8.3	7.0	2.3	0.4		4.4	6.3	10.5	1.1	0.1	0		24.0	22.4
	Model E	Sel	0	0.4	77.0	**16.2**	5.7	0.6	0.1	0	2.6	**87.5**	9.3	0.4	0	0.2		
		Pts	3.1	3.6	12.3	4.1	0.7	0.1		0	0.4	20.2	3.0	0.4	0.1		23.9	24.0
Scenario 3			0.01	0.03	0.10	**0.25**	0.34	0.47		0.01	0.03	0.07	0.15	**0.25**	0.37			
	Benchmark	Sel	0	0.3	14.6	**59.1**	22.0	4.0		0	0	2.1	26.7	**48.0**	23.1			
	Model A	Sel	0	0	34.9	**42.0**	21.2	1.9	0	0	0	13.4	39.5	**37.0**	8.9	1.2		
		Pts	3.0	3.0	8.3	6.9	2.6	0.2		1.2	2.3	5.9	8.5	4.7	1.1		24.0	23.7
	Model B	Sel	0	0.3	72.4	**22.0**	4.0	1.3	0	0	0.3	49.6	28.7	**15.5**	4.8	1.1		
		Pts	3.1	3.6	12.7	4.0	0.5	0.1		0.5	1.6	13.1	6.0	2.0	0.5		24.0	23.7
	Model C	Sel	0	0.3	79.4	**14.7**	4.8	0.7	0.1	0	0.2	54.2	25.9	**14.6**	4.6	0.5		
		Pts	3.2	3.6	12.5	4.0	0.6	0.1		3.3	3.4	10.2	5.3	1.4	0.4		23.9	23.9
	Model D	Sel	0	0.1	37.9	**43.0**	16.7	2.3	0	0	0.1	55.8	22.3	**15.2**	3.5	3.1		
		Pts	3.0	3.0	8.3	7.1	2.1	0.4		3.1	3.4	10.3	4.7	1.3	0.4		24.0	23.2
	Model E	Sel	0	0.4	77.0	**16.2**	5.7	0.6	0.1	0	0	69.9	21.2	**7.0**	1.7	0.2		
		Pts	3.1	3.6	12.3	4.1	0.7	0.1		0	0	18.0	4.6	1.1	0.4		23.9	24.0
Scenario 4			0.01	0.03	0.05	0.08	0.15	**0.25**		0.02	0.05	0.07	0.12	**0.25**	0.36			
	Benchmark	Sel	0.2	0	0.4	3.5	29.3	**66.6**		0	0.5	1.7	17.3	**56.4**	24.1			
	Model A	Sel	0	0	2.4	16.9	46.3	**34.4**	0	0	0	7.5	29.5	**44.5**	18.3	0.2		
		Pts	3.0	3.0	5.0	5.2	6.0	1.8		0.3	1.0	5.3	7.3	7.5	2.6		24.0	24.0
	Model B	Sel	0	0.1	28.4	26.1	28.7	**16.7**	0	0	0	36.8	32.9	**23.1**	6.3	0.9		
		Pts	3.1	3.4	8.0	5.7	2.7	1.1		0.2	0.8	10.0	7.8	3.6	1.3		24.0	23.7
	Model C	Sel	0	0.1	35.8	15.9	31.7	**16.3**	0.2		0.2	0.8	53.3	**21.5**	19.0	4.5	0.7	
		Pts	3.2	3.4	7.8	5.6	2.3	1.6		3.3	3.6	10.2	4.6	1.6	0.5		23.9	23.8
	Model D	Sel	0	0	4.1	16.4	45.3	**34.2**	0	0	1.2	49.1	24.6	**19.2**	4.9	1.0		
		Pts	3.0	3.0	4.6	5.4	5.1	2.9		3.4	3.6	9.7	5.0	1.6	0.5		24.0	23.8
	Model E	Sel	0	0.1	35.6	16.4	31.0	**16.8**	0.1	0	0	27.1	23.9	**37.7**	11.1	0.2		
		Pts	3.1	3.4	7.8	5.6	2.4	1.6		0	0	9.0	6.1	6.3	2.6		23.9	24.0
Scenario 5			**0.25**	0.34	0.47	0.55	0.65	0.75		0.40	0.50	0.60	0.70	0.80	0.90			
	Benchmark	Sel	**74.0**	21.9	3.8	0.2	0	0		98.4	1.4	0.2	0	0	0			
	Model A	Sel	**29.7**	32.4	16.3	0.1	0	0	**21.5**	0.5	0	0	0	0	0	**99.5**		
		Pts	6.2	6.2	6.7	0.2	0	0		0.1	0	0	0	0	0		19.3	0.1
	Model B	Sel	**26.1**	12.4	3.6	0	0	0	57.9	0.4	0.1	0	0	0	0	**99.5**		
		Pts	5.0	3.7	1.7	0.1	0	0		0.1	0.1	0	0	0	0		10.5	0.2
	Model C	Sel	**27.3**	11.8	3.1	0	0	0	**57.8**	0.8	0.1	0.1	0	0	0	**99.0**		
		Pts	5.1	3.7	1.6	0.1	0	0		0.2	0.1	0	0	0	0		10.5	0.2
	Model D	Sel	**30.4**	31.7	15.5	0.2	0	0	22.2	0	0	0	0	0	0	**100**		

Table S2 – *Continued.*

Design			% dose declared as MTD & average patient allocation														$\tilde{N}_{\mathcal{T}_1}$	$\tilde{N}_{\mathcal{T}_2}$
			Trial $\mathcal{T}_1$							Trial $\mathcal{T}_2$								
			d_{11} 0.1	d_{12} 0.5	d_{13} 1	d_{14} 5	d_{15} 10	d_{16} 20	None	d_{21} 0.1	d_{22} 0.5	d_{23} 1	d_{24} 5	d_{25} 10	d_{26} 20	None		
		Pts	6.2	5.7	7.1	0.2	0	0		0	0	0	0	0	0		19.2	0
	Model E	Sel	**26.6**	11.6	3.9	0	0	0	**57.9**	5.7	1.3	0.1	0	0	0	92.9		
		Pts	4.9	3.6	1.7	0.1	0	0		1.0	1.6	0.9	0	0	0		10.3	3.5
Scenario 6			0.01	0.03	0.05	0.08	0.15	**<u>0.25</u>**		0.10	**<u>0.25</u>**	0.36	0.50	0.60	0.68			
	Benchmark	Sel	0.2	0	0.4	3.5	29.3	**66.6**		14.7	**60.6**	22.2	2.5	0	0			
	Model A	Sel	0	0	2.8	15.3	49.7	**32.2**	0	16.2	**33.3**	38.8	2.3	0.2	0.1	9.1		
		Pts	3.0	3.0	4.8	5.4	6.0	1.8		3.0	5.2	11.6	1.7	0.4	0		24.0	21.9
	Model B	Sel	0	0.1	28.1	24.1	28.7	**18.9**	0.1	18.0	**30.1**	21.4	0.2	0	0	30.3		
		Pts	3.1	3.4	8.1	5.5	2.6	1.2		3.1	5.3	6.8	1.3	0.2	0.1		23.9	16.8
	Model C	Sel	0	0	37.8	18.1	28.2	**15.9**	0	26.4	**37.2**	22.3	0.6	0	0	13.5		
		Pts	3.2	3.4	8.1	5.6	2.2	1.5		7.5	7.5	5.5	0.3	0	0		24.0	20.8
	Model D	Sel	0	0	3.2	16.4	47.1	**33.3**	0	25.7	**37.2**	21.7	0.3	0	0	15.1		
		Pts	3.0	3.0	4.5	5.6	5.3	2.5		7.4	7.5	5.2	0.3	0	0		23.9	20.4
	Model E	Sel	0	0.1	35.6	16.4	31.0	**16.8**	0.1	0.6	**6.2**	71.0	20.6	1.4	0	0.2		
		Pts	3.1	3.4	7.8	5.6	2.4	1.6		0.1	0.4	13.8	7.0	2.5	0.3		23.9	24.0

pTox: true probability of toxicity in humans; **Sel**: proportion of times of declaring a dose as MTD; **Pts**: average number of patients allocated to a dose; **Benchmark**: Non-parametrical optimal benchmark design by O'Quigley *et al.* [1].

References

1. O'Quigley J, Paoletti X, Maccario J. Non-parametric optimal design in dose finding studies. *Biostatistics*. 2002; 3(1):51-56.

2. Zheng H, Hampson LV, Wandel S. A robust Bayesian meta-analytic approach to incorporate animal data into phase I oncology trials. *Statistical Methods in Medical Research*. 2020; 29(1):94-110.

3. Liu S, Pan H, Xia J, Huang Q, Yuan Y. Bridging continual reassessment method for phase I clinical trials in different ethnic populations. *Statistics in Medicine*. 2015;4(10):1681-1694.
